# Flowr.root – A flow matching based foundation model for joint multi-purpose structure-aware 3D ligand generation and affinity prediction

**Published:** 2025-11-06

**Authors:** Julian Cremer, Tuan Le, Mohammad M. Ghahremanpour, Emilia Sługocka, Filipe Menezes, Djork-Arné Clevert

**Affiliations:** 1 Machine Learning & Computational Sciences, Pfizer Worldwide R&D, Berlin, Germany; 2 Computational Chemistry, Medicine Design, Pfizer Worldwide R&D, Cambridge, USA; 3 Doctoral School of Medical and Health Sciences, Jagiellonian University Medical College, Cracow, Poland; 4 Institute of Structural Biology, Molecular Targets and Therapeutics Center, Helmholtz Munich, Neuherberg, Germany; 5 TUMSchool of Natural Sciences, Department of Bioscience, Bayerisches NMR Zentrum, Technical University of Munich, Garching, Germany; 6 Department of Physicochemical Drug Analysis, Faculty of Pharmacy, Jagiellonian University Medical College, Cracow, Poland

## Abstract

We present Flowr.root, an *SE*(3)-equivariant flow-matching model for pocket-aware 3D ligand generation with joint binding affinity prediction and confidence estimation. The model supports multiple design modes including *de novo* generation, interaction/pharmacophore-conditional sampling, fragment elaboration, and multi-endpoint affinity prediction (${\text{pIC}}_{50}$, $\text{p}{\text{K}}_{i}$, $\text{p}{\text{K}}_{d}$, ${\text{pEC}}_{50}$). Training combines large-scale ligand libraries with mixed-fidelity protein–ligand complexes, followed by refinement on curated co-crystal datasets and adaptation to project-specific data through parameter-efficient finetuning. Flowr.root achieves state-of-the-art performance in both unconditional 3D molecule and pocket-conditional ligand generation, producing geometrically realistic, low-strain structures with computational efficiency on established benchmark datasets. The integrated affinity prediction module demonstrates superior accuracy on the Spindr test set and outperforms recent models on the Schrödinger FEP+/OpenFE benchmark while offering substantial speed advantages. As a foundation model, Flowr.root requires continuous parameter-efficient finetuning on project-specific datasets to account for unseen structure-activity landscapes, which we demonstrate yields strong correlation with experimental in-house data. The model’s joint generation and affinity prediction capabilities enable inference-time scaling through importance sampling, effectively steering molecular design toward higher-affinity compounds. Case studies validate this approach: selective CK2α ligand generation against CLK3 shows significant correlation between predicted and quantum-mechanical binding energies, while scaffold elaboration studies on ERα, TYK2 and BACE1 demonstrate strong agreement between predicted affinities and QM calculations. By integrating structure-aware generation, affinity estimation, and property-guided sampling within a unified framework, Flowr.root provides a comprehensive foundation for structure-based drug design spanning hit identification through lead optimization.

## 1 Introduction

Diffusion- and flow-based generative models have emerged as leading frameworks in machine learning [Ho et al., 2020b, Song et al., 2021, Rombach et al., 2022]. Denoising Diffusion Probabilistic Models (DDPMs) learn a reverse process to transform noise into data samples [Ho et al., 2020b], while score-based models formulate generation via reverse-time stochastic differential equations (SDEs) or their probability-flow ordinary differential equation (ODE) counterparts [Song et al., 2021]. Flow matching, in turn, directly regresses the velocity field along a prescribed probability path to train continuous normalizing flows [Lipman et al., 2023, Liu et al., 2022], with stochastic-interpolant theory offering a unified perspective on diffusion and flow models [Albergo and Vanden-Eijnden, 2023, Albergo et al., 2023]. Beyond applications in natural language processing and computer vision, these models are increasingly applied to biology and chemistry, fueling advances in generative chemistry for drug discovery [Hoogeboom et al., 2022, Vignac et al., 2023, Le et al., 2023, Schneuing et al., 2023, Guan et al., 2023, Cremer et al., 2024, Campbell et al., 2024, Irwin et al., 2024, Cremer et al., 2025]. In drug discovery, designing small molecules that selectively bind to specific protein targets remains a critical challenge. Diffusion and flow models have proven effective at capturing complex molecular and structural distributions, advancing methods from 3D molecular generation in structure-based drug design (SBDD) to protein–ligand (co-)folding [Abramson et al., 2024, Passaro et al., 2025].

One important subfield of AI-driven SBDD is pocket-conditional, structure-aware ligand design. Models like DiffSBDD [Schneuing et al., 2023] and TargetDiff [Guan et al., 2023] have demonstrated that *SE*(3)-equivariant diffusion models can generate ligands directly within protein pockets. Follow-up studies have focused on improving chemical validity and pose accuracy [Le et al., 2023, Wang et al., 2024] while coupling pocket conditioning with large-scale pretraining and multi-objective importance sampling to guide generation toward potency and synthesizability [Cremer et al., 2024]. Additionally, AI-driven models have increasingly integrated fragment priors into generative pipelines [Imrie et al., 2020, Voloboev, 2024, Zhang et al., 2024, Lee et al., 2025, Guo et al., 2023, Igashov et al., 2024]. In fragment-based drug discovery (FBDD), which focuses on hit-to-lead optimization, models typically specialize either in *de novo* design or fragment-based strategies. However, practical lead optimization requires flexible navigation between these regimes, such as fragment growing or scaffold hopping under interaction constraints, which most current frameworks do not adequately address. Furthermore, many models do not incorporate the geometric constraints imposed by protein pockets. Recent work proposes a unified flow matching approach that integrates *de novo*, interaction-constrained, and fragment-based generation under pocket conditioning with efficient ODE sampling [Cremer et al., 2025].

However, the effectiveness of generated ligands ultimately depends on their potency, which requires reliable affinity prediction. While experimental validation remains indispensable, computational prioritization during the design phase relies on accurate affinity predictions to effectively navigate target-relevant regions of chemical space. Classical scoring functions, such as AutoDock Vina [Trott and Olson, 2010], Glide [Friesner et al., 2004], and GOLD [Jones et al., 1997], offer computational efficiency but often lack the necessary accuracy for reliable prioritization. Physics-based methods like free energy perturbation (FEP) and absolute binding free energy (ABFE) calculations provide higher precision [Wang et al., 2015, Mey et al., 2020, Mobley and Klimovich, 2012, Alibay et al., 2022, Feng et al., 2022a, Ries et al., 2024], yet their computational cost prohibits application to large-scale generative campaigns, restricting their use to small subsets of candidates where absolute affinity data is critical. Machine learning–based scoring functions improve throughput but suffer from dataset bias and limited generalization [Jiménez et al., 2018]. Recent approaches using data augmentation have shown modest improvements [Valsson et al., 2025], though they lack explicit structural information and remain insufficient for robust affinity prediction. The Boltz-2 ligand–protein co-folding model achieves near-FEP accuracy on selected targets with substantial speed advantages [Passaro et al., 2025]. However, its decoupled architecture—where the affinity head is trained independently from the structural module—creates dependence on structure prediction quality, requires co-folding prior to each affinity prediction, and may limit co-adaptation between protein pocket and ligand geometry, particularly during project-specific fine-tuning.

These limitations motivate a framework that jointly learns structure and affinity while supporting fast, controllable, structure-aware generation. A model that learns the joint probability distribution over ligand geometry, binding pose, and affinity within the protein pocket context would enable efficient, potency-guided ligand generation with on-the-fly ranking capabilities. Crucially, joint training facilitates simple project-specific adaptation through fine-tuning—an essential capability given the distinct constraints of drug discovery campaigns, ranging from ADME/T requirements to R-group and scaffold novelty constraints, diverse assay readouts, and medicinal chemistry heuristics. A fundamental challenge in structure-based generative modeling lies in the inherent disconnect between public-domain training data and project-specific structure-activity relationships (SARs). While models can achieve broad coverage of chemical space through ligand generation, generalizing across novel bioactivity landscapes represents a fundamentally more complex problem. We posit that expecting universal generalization without adaptation is unrealistic; instead, models should function as dynamic companions that continuously refine their understanding of project-specific SARs through sustained interaction with incoming data. This paradigm shift necessitates moving from static models to efficient iterative refinement processes where model utility grows through continuous adaptation. Additionally, techniques such as multi-objective guidance via inference-time importance sampling [Cremer et al., 2024] can help steering generation toward pre-specified, desired properties, while deeper distribution mismatches or activity cliffs can be addressed through direct preference alignment [Schneuing et al., 2025] when suitable data is available.

While large ligand-only resources such as ZINC and PubChem offer abundant chemical diversity [Irwin et al., 2020, Bolton et al., 2011], high-quality protein–ligand complexes with reliable affinity annotations remain scarce and noisy. Databases like PDBBind provide valuable training data [Wang et al., 2005], yet suffer from quality and coverage limitations; curated subsets such as HiQBind address these issues and even increase sample sizes [Wang et al., 2025], yet remain limited in scale. The Plinder dataset [Durairaj et al., 2024] expands scale by broadly curating the PDB, but most entries lack affinity annotations. More recent resources—BindingNet [Zhu et al., 2025], Kinodata-3D [Backenköhler et al., 2024], and especially SAIR [Lemos et al., 2025]—improve chemical space coverage and include affinity data, though they do so with reduced accuracy. This heterogeneous data landscape however, motivates a multi-stage training paradigm: by systematically combining available resources ranked by fidelity, we can exploit their complementary strengths. Large-scale, lower-fidelity data establishes broad chemical space coverage and foundational structural understanding, while subsequent refinement on higher-quality, curated datasets sharpens affinity prediction and structural accuracy. Critically, this approach yields foundation models capable of efficient adaptation to project-specific objectives.

In this work, we present Flowr.root, a foundational framework for structure-based generative chemistry designed to address the continuum from large-scale pre-training through project-specific adaptation. Flowr.root integrates *de novo*, interaction- and pharmacophore-, as well as fragment-conditioned ligand generation with multi-endpoint affinity prediction and per-sample confidence estimation. Built on the Flowr.MULTI backbone [Cremer et al., 2025], the model employs a three-stage training strategy: (1) large-scale pre-training on billions of ligands and millions of protein–ligand complexes with diverse affinity labels, (2) fine-tuning on curated, higher-fidelity datasets, and (3) project-specific adaptation via parameter-efficient fine-tuning and multi-objective guidance through importance sampling. Critically, Flowr.root jointly trains its confidence and affinity prediction modules with structure generation, enabling affinity to actively shape the generative trajectory and facilitating rapid adaptation to project-specific data distributions. This design supports the model’s intended role as a continuously evolving companion throughout early-stage drug discovery campaigns—assisting in motif identification, core expansion, and R-group design through sustained refinement on incoming structure-activity data.

## 2 Related Works

### Molecule generation

Early neural approaches to 3D molecular generation explored autoregressive models that sequentially add coordinates and atom types while maintaining geometric consistency. Symmetry-aware models such as G-SchNet [Gebauer et al., 2020] and its conditional inverse-design follow-up [Gebauer et al., 2022] demonstrated that enforcing *E*(3) symmetries and conditioning signals can substantially improve the validity and controllability of generated 3D structures. Subsequent work proposed explicit autoregressive flows [Luo and Ji, 2022]. In parallel, the diffusion modeling paradigm matured from its non-equilibrium thermodynamics roots [Sohl-Dickstein et al., 2015] through score-based [Song et al., 2021] and variational formulations [Ho et al., 2020a, Kingma et al., 2021]. These ideas rapidly translated to molecular geometry: Xu et al. [2022] and Jing et al. [2023] showed that *E*(3)-equivariant denoisers enable high-quality conformation generation by diffusing in Cartesian and torsional spaces, respectively.

The first *E*(3)-equivariant diffusion model for joint continuous coordinate and atom type generation was EDM [Hoogeboom et al., 2022]. Follow-ons pushed the design space along several axes: discrete and continuous combination with explicit bond order learning [Vignac et al., 2023], and enhanced denoising learning objectives [Le et al., 2023], establishing strong baselines.

Thereafter, diffusion-based methods pioneered pocket-aware *de novo* design, also utilizing *E*(3)-equivariant networks. Notably, DiffSBDD [Schneuing et al., 2023] and TargetDiff [Guan et al., 2023] demonstrated that conditional diffusion models can generate diverse, target-specific ligands within the protein pocket, adhering to symmetry constraints and enabling task versatility through sampling controls. More recently, Pilot [Cremer et al., 2024] combined large-scale pretraining, pocket conditioning, and property guidance, highlighting the importance of multi-objective steering (e.g., drug-likeness, synthesizability) under structure constraints. Together, these works established that pocket-aware 3D diffusion can simultaneously respect symmetry, improve pose realism, and support versatile constraints via conditioning and guided sampling.

Meanwhile, flow matching [Lipman et al., 2023] (FM) was proposed refining continuous normalizing flows by directly regressing a time-dependent velocity field that pushes a simple prior to the data distribution, offering faster sampling and flexible priors. *SemlaFlow* [Irwin et al., 2024] introduced a scalable *SE*(3)-equivariant architecture (Semla) trained via flow matching, achieving state-of-the-art unconditional 3D molecule generation with significant speed-ups. Pushing FM into SBDD, Flowr [Cremer et al., 2025] extended the Semla-style backbones with an dedicated pocket encoder and mixed continuous/categorical FM, supporting multi-mode *de novo*, interaction-guided, and fragment-based generation in a single model. Flowr reports large speedups over pocket-diffusion baselines.

Fragment-based drug discovery (FBDD) motivates models that initiate from fragments and perform growth, linking, or merging under pocket constraints. Early deep generative linker design incorporated 3D information into graph models (DeLinker) Imrie et al. [2020], while SyntaLinker and AutoLinker utilized conditional transformers to synthesize linkers directly in the SMILES space, given fragment pairs and constraints Yang et al. [2020b], Feng et al. [2022b].

Recent advances have introduced *E*(3)-equivariant models: DiffLinker formulates linker generation as an *E*(3)-equivariant conditional diffusion, explicitly learning 3D geometry between fragment anchors Igashov et al. [2024]. For broader medicinal-chemistry workflows, Link-INVENT extends REINVENT with reinforcement learning to optimize linkers for multiple objectives, demonstrated on fragment linking, scaffold hopping, and PROTAC design Guo et al. [2023]. STRIFE extracts target-specific pharmacophoric features to steer elaboration in 3D Hadfield et al. [2022], while AutoFragDiff integrates fragment-wise, autoregressive diffusion with pocket conditioning to improve local 3D geometry during growth Ghorbani et al. [2023]. For scaffold hopping, DiffHopp employs an *E*(3)-equivariant graph diffusion model tailored for scaffold replacement conditioned on a protein–ligand complex Torge et al. [2023], and TurboHopp accelerates pocket-conditioned 3D scaffold hopping with consistency models and reinforcement learning-based preference optimization Yoo et al. [2024].

### Binding Affinity Prediction

Estimating the change in free energy upon binding ($\text{Δ}G_{\text{bind}}$, or affinity) accurately remains a cornerstone of structure-enabled small-molecule discovery. Binding affinity is relevant for all early stages of drug discovery, starting from hit identification, where the goal is to find tight and selective binders, through hit-to-lead and lead optimization, where potency must be balanced with absorption, distribution, metabolism, excretion, safety, toxicity, and efficacy considerations. Given the astronomical size of chemical space, computer-aided drug design (CADD) is indispensable to select and prioritize candidates *in silico* before spending scarce experimental resources [Reymond et al., 2010, Jorgensen, 2004]. Classical structure-based approaches to affinity prediction span knowledge-based scoring and physics-based models grounded in molecular mechanics [Liu and Wang, 2015, Gilson and Zhou, 2007]. Heuristic docking scores offer speed at the expense of physical rigor. Empirical scoring functions such as AutoDock Vina, Glide, or GOLD remain widely used due to speed, but show inconsistent results between targets Trott and Olson [2010], Friesner et al. [2004], Jones et al. [1997]. Semi-empirical and QM / MM scoring have closed part of the gap at an intermediate cost, for example, SQM2.20 achieves DFT-quality affinity estimates in minutes, but only on selected targets [Pecina et al., 2024, Molani and Cho, 2024]. End-point methods such as MM-PBSA and MM-GBSA combine molecular mechanics with continuum solvation to approximate the $\text{Δ}G_{\text{bind}}$ from MD snapshots at a relatively low cost and remain widely used when throughput is critical [Kollman et al., 2000, Homeyer and Gohlke, 2012, Still et al., 1990, Gohlke and Case, 2004]. Alchemical binding free energy methods, absolute (ABFE) and relative (RBFE), trade throughput for accuracy [Aldeghi et al., 2016, Boresch et al., 2003, Gilson et al., 1997, Fu et al., 2022, Feng et al., 2022a, Cournia et al., 2017, Ross et al., 2023a]. Modern workflows based on the free energy perturbation theory (FEP) [Zwanzig, 1954] have achieved impressive accuracy on suitable congeneric series [Abel et al., 2017, Wang et al., 2015, Ross et al., 2023b], but remain sensitive to force fields and system preparation.

Machine learning (ML) offers a complementary path to rapid affinity estimation by learning structure–activity relationships directly from data. The early ML scoring functions used interaction fingerprints and hand-made descriptors [Ballester and Mitchell, 2010, Wójcikowski et al., 2019, Kundu et al., 2018, Boyles et al., 2020]. Sequence-based CPI / DTA models (e.g., DeepDTA) encode proteins and ligands from 1D inputs to predict binding affinity [Özturk et al., 2018], while more recent deep architectures, such as 3D convolutional neural networks and graph neural networks, operate more holistically on complex geometry and interaction graphs [Jiménez et al., 2018, Jiang et al., 2021, Karlov et al., 2020, Nguyen et al., 2021, Li et al., 2021, Meli et al., 2022, Valsson et al., 2025].

ML models are typically trained and evaluated on community benchmarks (e.g., CASF) [Li et al., 2018, Su et al., 2019]. However, strong in-benchmark performance does not guarantee generalization. Multiple analyses show that models can overfit ligand biases, struggle on out-of-distribution (OOD) targets, or even partially fit to noise [Volkov et al., 2022, Scantlebury et al., 2023, Yang et al., 2020a, Crusius et al., 2025]. This limits their reliability in prospective campaigns and underscores the need for approaches that encode biophysical constraints, reduce dataset shortcuts, and validate on OOD benchmarks. Compounding these challenges is data scarcity: structure-based learning ideally requires reliable affinity measurements paired with high-resolution 3D protein–ligand complexes. Although data augmentation is a mainstay in computer vision and NLP [Paulin and Ivašić-Kos, 2023, Pellicer et al., 2023], generating meaningful molecular data that respect stereochemistry, conformational physics, and pocket geometry remains non-trivial. However, combining ChEMBL and PDBBind through comparative complex structure and enhanced template-based modeling resulted in the BindingNet resource, comprising *ca.* 690k complexes. This significantly densifies the bioactivity landscape compared to the PDBBind alone [Li et al., 2024, Zhu et al., 2025]

A recent advancement is Boltz-2, a co-folding foundation model that predicts complex structures and based on that binding affinity, approaching FEP-level accuracy on certain targets while running orders of magnitude faster, makes large-scale affinity ranking feasible [Passaro et al., 2025]. Boltz-2’s affinity module couples structural inference with potency prediction, providing a stronger supervisory signal than *post hoc* scoring and highlighting the value of unified structure–affinity modeling in end-to-end pipelines[Passaro et al., 2025].

## 3 Datasets

To comprehensively train and evaluate Flowr.root for structure-aware ligand design, we leverage a diverse collection of public datasets spanning both small molecules and biomolecular complexes. Our dataset selection encompasses three primary categories: (1) large-scale small molecule databases for training conformational and chemical diversity, (2) large-scale computationally generated datasets that bridge the gap between available experimental data and the scale and augmentation required for deep learning applications, and (3) small-scale experimental protein-ligand complex datasets for higher-fidelity structure-based modeling. This multi-faceted approach ensures robust model training across diverse chemical, conformational, and biological/bio-activity spaces while maintaining high structural quality standards.

**Figure 1:**
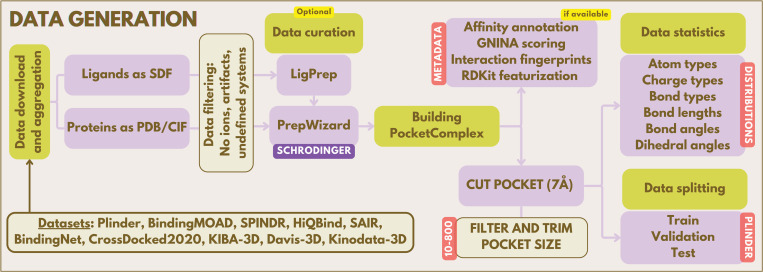
Overview of the dataset generation pipeline used in this work. Dataset generation workflow comprising data filtering, curation via Schrodinger’s LigPrep and PrepWizard, building of metadata-annotated internal representation, and calculation of molecule statistics.

For our small molecule database, we utilized Zinc3D, PubChem3D, Enamine REAL, and OMol25. For our protein-ligand database, we assembled a comprehensive collection of protein–ligand complex datasets by aggregating and standardizing data from multiple sources, including Plinder, BindingMOAD, Spindr, HiQBind, SAIR, BindingNet, CrossDocked2020, KIBA-3D, Davis-3D, and Kinodata-3D. As visualized in Fig. 1, after rigorous preprocessing, filtering, and preparation of both ligands and proteins, each complex was converted into a unified internal representation and annotated with extensive metadata, such as affinity values, if available, and molecular descriptors. This harmonized dataset enables systematic analysis of chemical composition, structural diversity, and affinity distributions across all included sources, providing a robust foundation for downstream modeling and benchmarking.

While Spindr and HiQBind provide preprocessed and well-curated co-crystal data resources, we applied additional comprehensive curation to selected datasets, namely Plinder, BindingMoad, SAIR, KIBA-3D and Davis-3D using Schrödinger’s LigPrep and PrepWizard tools. We used LigPrep to generate multiple protonated molecular conformations considering among other things different tautomeric states at physiological pH (7.4 ± 2.0), utilizing the OPLS4 force field and Epik for accurate $\text{p}K_{a}$ prediction aligned with the reference ligand via the maximum common substructure (MCS). PrepWizard handled protein preparation through side chain completion, protonation state determination using Epik and PROPKA, termini capping, water molecule sampling within 10.0 Å, constrained hydrogen and overall restrained minimization (0.3 Å RMSD tolerance) using the S-OPLS force field. Unless otherwise stated, protein pockets were extracted using a 7Å cutoff radius around the respective reference ligands, with constraints of a minimum of 10 and maximum of 800 pocket atoms per complex to ensure computational tractability while preserving essential binding site information.

Importantly, throughout all protein-ligand datasets we kept a consistent dataset split following the provided Plinder [Durairaj et al., 2024] train, validation and test set splits to avoid data leakage as best as possible enabling a stringent downstream evaluation. For more details about the datasets we refer to App. A, where we provide an overview of the aforementioned dataset generation, curation, and preprocessing pipeline, and a set of different dataset statistics.

### Zinc3D

Zinc3D [Irwin et al., 2020] is a subset of the ZINC20 database containing pre-computed low-energy conformations for commercially available compounds. By providing ready-to-use 3D structures, ZINC3D eliminates the computational overhead of on-the-fly conformer generation, thereby accelerating virtual screening campaigns and structure-based drug discovery workflows. We utilized 646,663,126 molecules with their provided conformations.

### PubChem3D

PubChem3D Bolton et al. [2011] extends the widely-used PubChem database by providing up to 500 computed 3D conformations for millions of bioactive compounds. This resource offers extensive conformational diversity, crucial for training robust generative models. We employed 10 conformations per molecule from approximately 93 million compounds, resulting in 928,649,525 total conformations.

### Enamine REAL

The Enamine REAL database comprises commercially available, synthetically accessible compounds widely employed in virtual screening and drug design. Its well-curated chemical space supports generative design strategies focused on drug-likeness and synthetic feasibility. Following Cremer et al. [2024], we used a diversity subset of the Enamine REAL database. We employed OpenEye’s Omega software with default parameters to generate up to five conformers per molecule, yielding 111,389,149 conformations.

### OMol25

OMol25 [Levine et al., 2025] is a comprehensive dataset containing over 100 million density functional theory (DFT) calculations at the *ω*B97M-V/def2-TZVPD level of theory. The dataset covers systems up to 350 atoms with exceptional chemical and structural diversity across 83 elements. We utilized two subsets: the small molecules collection (21,352,259 structures) comprising recomputed versions of widely-used datasets (ANI-2X, Orbnet Denali, SPICE2, Solvated Protein Fragments, and 30% of GEOM) upgraded to consistent high-level DFT theory, and the biomolecules subset (5,180,233 structures), encompassing protein-ligand interactions derived from BioLiP2 and additional complexes generated through docking drug-like molecules from GEOM, ChEMBL, and ZINC20. The biomolecule dataset features protein pocket environments processed through molecular dynamics simulation with appropriate capping and protonation state sampling.

### KIBA-3D

We introduce KIBA-3D, a novel kinase-focused dataset derived from the KIBA bioactivity dataset Tang et al. [2014a]. Using Schrödinger’s Glide, we performed exhaustive cross-docking of the KIBA ligand space across 172 kinase targets, generating 333,670 protein–ligand complexes spanning 2,038 unique ligands. This dataset provides a dense bioactivity landscape ideally suited for affinity guided, pocket-conditioned generative modeling and kinase-specific benchmarking applications.

### Davis-3D

DAVIS-3D is a structurally augmented version of the original DAVIS kinase bioactivity dataset [Davis et al., 2011], created as part of the Folding-Docking-Affinity (FDA) framework [Wu et al., 2025]. The dataset was generated by computationally folding protein structures using ColabFold and determining protein-ligand binding conformations through DiffDock, a deep learning-based docking model. This process transforms the original sequence-based DAVIS dataset into a collection of three-dimensional protein-ligand binding structures, of which we utilized 12,982 complexes with binding affinities.

### Kinodata-3D

Kinodata-3D [Backenköhler et al., 2024] is a curated collection of kinase complexes processed using cross-docking methodologies. We utilized 94,211 complexes from the combined mid- to higher-confidence subsets, which include binding affinity annotations and are preprocessed with a 5Å protein-ligand pocket cutoff.

### BindingNet

BindingNet [Li et al., 2024, Zhu et al., 2025] comprises 689,796 modeled protein-ligand binding complexes across 1,794 protein targets. The dataset was constructed using an enhanced template-based modeling workflow that incorporates pharmacophore and molecular shape similarities alongside chemical similarity. Structures are categorized by template matching quality into high-confidence (232,030), moderate-confidence (164,912), and low-confidence (292,813) subsets, all of which we employed with their respective binding affinity annotations.

### Plinder

Plinder [Durairaj et al., 2024] represents the largest and most comprehensively annotated protein-ligand interaction (PLI) dataset, containing 449,383 PLI systems with over 500 annotations per complex. The dataset encompasses diverse interaction types including multi-ligand systems, oligonucleotides, peptides, and saccharides. Plinder introduces a novel approach for generating training and evaluation splits that minimizes task-specific leakage while maximizing test set quality. After removing complexes containing ligand artifacts, ions, or undefined ligands, and processing using Schrödinger’s LigPrep and PrepWizard, we utilized 250,633 protein-ligand systems.

### SAIR

The Structurally Augmented IC50 Repository (SAIR) [Lemos et al., 2025] is the largest publicly available dataset of protein-ligand 3D structures with binding affinity annotations, addressing the scarcity of high-quality experimental structures for deep learning applications. The original dataset contains 5,244,285 computationally generated structures across 1,048,857 unique protein-ligand systems from ChEMBL and BindingDB, with structures folded using the Boltz-1x model [Wohlwend et al., 2024]. We applied stringent filtering criteria, retaining only PoseBusters-valid [Buttenschoen et al., 2024] complexes with negative AutoDock-Vina scores [Eberhardt et al., 2021], confidence scores ≥0.8, interaction PTM ≥0.6, and IPTM ≥0.8, yielding 1,781,634 complexes. Further processing using Schrödinger’s LigPrep and PrepWizard resulted in 1,564,677 curated complexes with IC50 annotations.

### BindingMOAD

BindingMOAD [Hu et al., 2005] is a comprehensive database developed over two decades (2001–2025), containing 41,409 protein-ligand complexes with affinity coverage for 15,223 complexes (37%) and 20,387 unique ligands. After preprocessing with Schrödinger’s LigPrep and PrepWizard, we obtained 33,286 complexes with diverse binding affinity annotations.

### Spindr

Spindr [Cremer et al., 2025] is a higher-fidelity dataset of protein–ligand complexes curated for interaction-aware modeling. The dataset emphasizes accurate binding site geometries and ligand poses, supporting physically consistent generative modeling. Complexes are thoroughly cleaned to focus on drug-like, non-covalent interactions using Schrödinger’s PrepWizard, with protein-ligand interactions annotated using ProLIF [Bouysset and Fiorucci, 2021]. We employed all 35,627 provided complexes with partial binding affinity annotations.

### HiQBind

HiQBind [Wang et al., 2025] is a curated dataset addressing structural artifacts in widely-used datasets, like PDBbind. Containing over 18,000 unique PDB entries and 30,000 protein-ligand complex structures, it matches binding free energies from BioLiP, Binding MOAD, and BindingDB with co-crystallized PDB complexes. The dataset employs strictly open-source curation tools with multiple quality control modules for steric clash detection, ligand structure fixing, protein completion, and hydrogen addition protocols. We utilized all 31,571 complexes with binding affinity annotations.

### Schrodinger FEP+ dataset

The Schrodinger FEP+ dataset [Ross et al., 2023b] comprises a large and diverse collection of protein-ligand complexes, each featuring congeneric series of small molecules with experimentally measured binding affinities (${\text{K}}_{d}$, ${\text{K}}_{i}$, or ${\text{IC}}_{50}$). Designed as a benchmark for assessing the accuracy and reproducibility of free energy perturbation (FEP) methods, the dataset emphasizes high-quality structural data, including X-ray structures and carefully curated protein and ligand preparations. It covers a broad chemical space, including challenging cases such as macrocycles, charge-changing transformations, and buried water displacement. This dataset is intended to support the development, validation, and comparison of computational methods for predicting relative binding affinities, providing a robust foundation for downstream applications in drug discovery and molecular design.

## 4 Methods

This section outlines the methodology employed in our framework, Flowr.root, for generative structure-aware ligand modeling and binding affinity prediction. We provide a detailed description of the architecture and key components, including framework details about the three-stage training process, the generative modes available for ligand design, and the affinity prediction capabilities. We also discuss the strategies for confidence estimation, inference-time scaling via importance sampling, and domain adaptation as main application in practical drug discovery workflows.

### 4.1 Flowr.root

Flowr.root is built on the recently proposed Flowr architecture, an *SE*(3)-equivariant flow matching model that learns a mixed continuous/discrete transport map. This map transforms a prior distribution (e.g., random noise or fragment anchors for coordinates) to the target ligand distribution within a given protein pocket [Cremer et al., 2025]. The model consists of two main components: a pocket encoder and a ligand decoder.

Protein pockets are extracted from holo structures by cutting residues within a defined radius around the binding site. These pockets are then encoded using an equivariant self-attention module followed by equivariant feed-forward layers, generating a set of invariant and equivariant protein features [Cremer et al., 2025]. The ligand decoder processes noisy ligand coordinates, partial charges, atom types, bond orders, and hybridization states through equivariant self-attention modules, which capture intra-ligand dependencies. A cross-attention layer then integrates contextual information from the pocket features to the hidden ligand features. For additional details, we refer to Cremer et al. [2025].

In Flowr.root, the ligand decoder has three output heads: (1) a structure head that predicts atomic coordinates, atom types, bond orders, charges, and hybridization states; (2) an affinity head that estimates four binding affinity types—${\text{IC}}_{50}$, $\text{p}K_{i}$, $\text{p}K_{d}$, and ${\text{pEC}}_{50}$; and (3) a confidence head that provides uncertainty estimation based on pLDDT [Abramson et al., 2024].

This multi-task formulation ensures that structural accuracy, potency estimation, and confidence calibration occur simultaneously during ligand generation. Following Cremer et al. [2025], Flowr.root also supports three generative modes within a single backbone: (1) *de novo* generation, (2) interaction-guided generation (to preserve or enforce pharmacophoric patterns), and (3) fragment-based elaboration. The latter mode uses fragments as anchors to generate new structures that are consistent with the binding pocket, enabling fragment growing, merging, linking, and scaffold hopping/elaboration. This multi-mode capability allows flexible applications in hit identification to fragment-based lead optimization.

The core challenge in protein-ligand modeling, however, lies not just in generation, but in accurately predicting protein-ligand interactions and potency. The sparsity of experimental binding data—where most ligand–protein combinations remain unexplored—poses a significant obstacle to improving these predictions. While broad-coverage datasets encompass a wide spectrum of protein targets, they are intrinsically sparse, with few ligands tested systematically across different biological contexts. This imbalance leads to biased datasets, which explains why 3D machine and deep learning models—often relying only on protein or ligand descriptors and ignoring interaction patterns—can perform surprisingly well on affinity prediction benchmarks like PDBBind. These inflated results also reveal systematic biases in the data, with activity clusters within certain protein families or chemical series [Volkov et al., 2022].

Real-world drug discovery requires understanding nuanced structure–activity relationships (SAR) across hundreds or even thousands of related molecules within the same binding site—a richness uniquely captured by dense bioactivity landscapes. These landscapes encode redundancy and critical phenomena like activity cliffs, where small structural changes can lead to significant shifts in bioactivity due to interaction changes.

This motivates the multi-stage training approach of Flowr.root: starting with sparse, lower-fidelity data to learn broad 3D chemistry and binding principles, and progressively refining the model with denser, higher-fidelity and project-specific datasets that capture subtle SAR patterns essential for effective hit expansion to lead optimization. This multi-stage pipeline is intended to closely mirrors the practical needs of early drug discovery campaigns, addressing the limitations imposed by data sparsity in current 3D affinity prediction models.

In summary, the Flowr.root framework consists of three stages:

#### Stage 1: Large-scale pre-training.

This stage builds a general-purpose generative prior across chemical and structural distributions, with a broad bioactivity landscape, albeit with varying quality and predictive relevance. The model is trained on a mixture of:

- **Small molecules in vacuum** (∼1.5B datapoints from ZINC3D, PubChem3D, Enamine, OMol25) to ensure broad chemical coverage.
- **Protein–ligand complexes with and without affinity labels** (∼2.5M datapoints from Plinder, BindingMoad, BindingNet, SAIR, KIBA-3D, Kinodata-3D, OMol25), which capture protein-ligand structural diversity, in parts annotated with affinity data. This dataset is split into lower- and higher-fidelity categories. We classify complexes with computational origin (i.e., BindingNet, SAIR, KIBA-3D, Kinodata-3D) as lower-fidelity and those stemming from co-crystal data (i.e., Plinder, BindingMoad) as higher-fidelity, with the exception of the high-confidence subset of BindingNet, which is treated as higher-fidelity.

#### Stage 2: High-fidelity fine-tuning.

Pre-trained weights are adapted to thoroughly curated, drug-like, high-quality affinity datasets. In this work, we use Spindr and HiQBind (∼30k complexes) as datasets for multi-target fine-tuning. However, the main goal of this stage is to fine-tune Flowr.root on high-quality in-house data, which teaches the model the fine details of protein-ligand interactions and binding affinity.

#### Stage 3: Project-specific domain adaptation.

This ensures that Flowr.root can bridge the gap between large-scale, noisy public data and small-scale, high-quality, in-assay data, producing distributions that align with specific discovery campaigns. Different domain adaptation strategies are used to fine-tune and steer the pre-trained Flowr.root model:

- **Inference-time scaling and property steering:** Importance sampling and path reweighting guide the model toward desired properties such as affinity, ADME/T, or synthetic accessibility.
- **Direct preference alignment:** The model is fine-tuned with project-specific preferences, such as avoiding known liability motifs or penalizing activity cliffs (future work).
- **Standard fine-tuning:** Parameter-efficient fine-tuning, with the freezing of non-critical modules, helps avoid catastrophic forgetting while aligning the model to project-specific structure-activity modes.

### 4.2 Training and Inference

Closely following the methodology of Cremer et al. [2025], we adopted the training and parameterization scheme from Flowr.MULTI. Specifically, we employed a 4-layer pocket encoder with $d_{\text{inv}}^{\text{enc}}=256$ and a 12-layer ligand decoder with $d_{\text{inv}}^{\text{dec}}=384$. The equivariant feature dimension was set to $d_{\text{equi}}=128$ for both the pocket encoder and ligand decoder. For latent attention, we utilized a latent size of 64 with 32 attention heads.

To further enhance model stability and generalization, we incorporated residual coordinate and subsequent edge updates using Gaussian radial basis expansions [Le et al., 2023], applied to the layer-wise latent message-passing outputs of the Flowr model backbone [Cremer et al., 2025]. Additionally, we included distance and cross-product computations between latent equivariant node features within the message-passing layers, which we found to improve model performance. Specifically, in the pairwise interactions between nodes, given the equivariant queries $q_{\text{equi}}\in {\mathbb{R}}^{B\times N_{q}\times 3\times d_{\text{equi}}}$ and keys $k_{\text{equi}}\in {\mathbb{R}}^{B\times N_{kv}\times 3\times d_{\text{equi}}}$, we define the pairwise difference tensor as

D=Q[:,:,1,:]−K[:,1,:,:],

and compute the distance features as

$$S=|D{|}_{2}\in {\mathbb{R}}^{B\times N_{q}\times N_{k}\times d}.$$

The pairwise cross product tensor is defined as

C=Q[:,:,1,:]×K[:,1,:,:],

where × denotes the vector cross product over the 3-vector dimension, resulting in

$$C\in {\mathbb{R}}^{B\times N_{q}\times N_{k}\times 3\times d}.$$

Both S and P are stacked to the existing pairwise message tensor as additional features.

The model structure output comprises predictions for atomic coordinates, atom types, charge, hybridization, and bond types. The overall loss function is:

$$L_{total}=\lambda_{c}\underset{\mathrm{Coordinate loss}}{\underset{︸}{\text{MSE}\left(X_{pred},X_{true}\right)}}+\lambda_{t}\underset{\mathrm{Atom type loss}}{\underset{︸}{\text{CE}\left(T_{pred},T_{true}\right)}}+\lambda_{\mathrm{ch}}\underset{\mathrm{Charge loss}}{\underset{︸}{\text{CE}\left(Q_{pred},Q_{true}\right)}}+\;\;\lambda_{h}\underset{\mathrm{Hybridization loss}}{\underset{︸}{\text{CE}\left(H_{pred},H_{true}\right)}}+\lambda_{b}\underset{\mathrm{Bond type loss}}{\underset{︸}{\text{CE}\left(B_{pred},B_{true}\right)}}$$

with 33.8M trainable parameters.

Flowr.root jointly models both continuous (coordinates) and discrete (atom types, bond orders, charges, hybridizations) molecular features. For coordinates, we employ continuous flow matching [Tong et al., 2024], while discrete flow models [Campbell et al., 2024] are used for categorical variables. The model is trained to recover the original ligand $l_{1}$ from a noisy ligand $l_{t}$ by learning the conditional distribution $p_{1|t}^{\theta }\left(l_{1}|l_{t},t;P\right)$, minimizing mean squared error for coordinates and cross-entropy loss for categorical features. Given a pocket structure P, novel ligands are generated by iteratively refining an initial noisy ligand $l_{0}\sim p_{\text{noise}}$. The generative process follows a learned vector field $v_{t}^{\theta }$ for continuous features and a discrete integration scheme for categorical attributes [Campbell et al., 2024, Cremer et al., 2025].

#### Binding affinity prediction

In drug discovery, a variety of parameters, including ${\text{K}}_{i}$, ${\text{K}}_{d}$, ${\text{IC}}_{50}$, and ${\text{EC}}_{50}$, are reported as potency measures. These parameters differ in their definitions and experimental setups, which complicates their direct comparison. For example, ${\text{K}}_{d}$ is an equilibrium constant that directly measures the strength with which a ligand binds to its target protein. On the other hand, a ${\text{K}}_{i}$ is an equilibrium constant indicating how well a given inhibitor inhibits the binding of a natural substrate. In other words, while ${\text{K}}_{d}$ measures the ”stickiness” of a molecule to a target, ${\text{K}}_{i}$ measures how well the inhibitor blocks the enzyme from its natural substrate. Though these values often correlate, they are not always interchangeable. Likewise, ${\text{EC}}_{50}$ and ${\text{IC}}_{50}$ are used for biological assays. Though they correlate with ${\text{K}}_{d}$ and ${\text{K}}_{i}$, their interpretation depends on assay conditions.

Therefore, we predict each potency measure separately, aligning better with the complexity of drug-target data. Given invariant and equivariant ligand features $h_{i}^{\text{inv}}$, $h_{i}^{\text{equi}}$, pocket features $p_{j}^{\text{inv}}$, $p_{j}^{\text{equi}}$, and ligand-pocket interaction features $e_{ij}$ extracted from the ligand decoder’s latent message-passing module, the binding affinity head computes:

$$\;f_{i}^{\text{lig}}=\mathrm{Gate}\left(h_{i}^{\text{inv}},h_{i}^{\text{equi}}\right)\;\;z^{\text{lig}}={\text{MLP}}_{\text{lig}}\left(\left[\frac{1}{C}\sum_{i}f_{i}^{\text{lig}},\frac{1}{\left|L\right|}\sum_{i}f_{i}^{\text{lig}}\right]\right)\;f_{j}^{\text{pocket}}=\mathrm{Gate}\left(p_{j}^{\text{inv}},p_{j}^{\text{equi}}\right)\;z^{\text{pocket}}={\text{MLP}}_{\text{pocket}}\left(\frac{1}{\left|P\right|}\sum_{j}f_{j}^{\text{pocket}}\right)\;\;z^{\text{int}}={\text{MLP}}_{\text{int}}\left(\frac{\sum_{i,j}e_{ij}m_{i}m_{j}}{\sum_{i,j}m_{i}m_{j}}\right)$$

where Gate is a Gated equivariant block [Schütt et al., 2021] to combine equivariant and invariant feature tensors, and MLP is a multi-layer perceptron with two linear layers and SiLU activation. The input to ${\text{MLP}}_{\text{lig}}$ is the concatenation of normalized invariant features, where C=100, and |L| denotes the number of atoms in the ligand. The concatenated feature tensor $z=\left[z^{\text{lig}},z^{\text{pocket}},z^{\text{int}}\right]$ is passed to task-specific heads:

$$y_{\text{affinity}}=\mathrm{ReLU}\left({\mathrm{MLP}}_{\text{affinity}}(z)\right)$$

The affinity prediction loss is computed using the mean Huber loss between predicted and true affinity values in log units (${\text{pIC}}_{50}$, ${\text{pK}}_{d}$, ${\text{pK}}_{i}$, ${\text{pEC}}_{50}$):

(1) $$L_{\text{affinity}}=\frac{1}{N}\sum_{i=1}^{N}\mathrm{Huber}\left({\hat{y}}_{i},y_{i}\right)$$

where ${\hat{y}}_{i}$ and $y_{i}$ are the predicted and true affinity values for valid samples i, and N is the number of valid samples.

To convert affinity values to experimental free energy ΔG values for comparison with physics-based models, we use the following formula:

(1) ΔG=−RTln10⋅pK

where $R=1.987\times 10^{-3}$ kcal K^−1^ mol^−1^ is the universal gas constant and T=298 K is the absolute temperature and pK being the respective affinity type.

#### Confidence prediction: Predicted local distance difference test (pLDDT)

We also predict pLDDT confidence scores to assess the reliability of generated ligand structures. Given predicted ligand samples ${\hat{l}}_{1}=(\hat{X},\hat{T},\hat{Q},\hat{H},\hat{B})$ and ground-truth ligand-protein coordinates $X_{l}\in {\mathbb{R}}^{N_{l}\times 3}$ and $X_{p}\in {\mathbb{R}}^{N_{p}\times 3}$, we compute distance matrices D, $\hat{D}\in {\mathbb{R}}_{+}^{N_{l}\times N_{p}}$ for true and predicted ligand-protein distances, respectively. We consider distances below 12.0Å with $b_{i}=\mathrm{clamp}\left(\sum_{j=1}^{N_{p}}\left(D_{i,j}<12.0\right),\min=1\right)$ neighbors for each ligand atom.

Using distance thresholds $\tau =\left\{\frac{1}{2},1,2,4\right\}$ and L1 distance errors $L=|D-\hat{D}|$, the LDDT score for each ligand atom is:

$${\mathrm{LDDT}}_{i}=\frac{1}{b_{i}}\sum_{j=1}^{N_{p}}\left[\frac{1}{\left|\tau \right|}\sum_{c\in \tau }\left(L_{ij}<c\right)\right]\in (0,1)$$

Each atom’s LDDT score is binned into k=50 categories for multi-class classification. The confidence head, $f_{\phi }$, shares the ligand decoder backbone with reduced depth (l=8 layers), taking the generated structure ${\hat{l}}_{1}$ as input and outputting invariant logits ${\text{pLDDT}}_{i}\in {\mathbb{R}}^{50}$ for cross-entropy loss minimization.

### 4.3 Inference-time scaling

Inference-time scaling is a powerful approach that allocates additional computation during generation, rather than solely relying on larger architectures [Snell et al., 2024, Setlur et al., 2024]. Originally developed for large language models, this approach has been extended to diffusion and flow matching models. Diffusion models leverage stochasticity through particle sampling [Wu et al., 2023, Cremer et al., 2024] and Feynman-Kac steering [Singhal et al., 2025], where multiple trajectories are generated and resampled based on reward functions. Flow matching models, despite being deterministic, achieve inference-time scaling through SDE-based generation, interpolant conversion, and Rollover Budget Forcing (RBF) for adaptive resource allocation [Kim et al., 2025], improving upon diffusion-based advances [Ma et al., 2025].

In our work, we steer generative trajectories to sample from the conditional (target) distribution

$$p_{\phi }(l|y,P)\propto r(y|l,P)p_{\theta }(l|P),$$

where y is a vector of properties such as binding affinity, and r represents a reward function that encodes user preferences.

#### Steering via Importance Sampling

The target distribution is usually intractable, especially when applying neural generative models that transport samples over time, such as flow or diffusion models. One way to sample from the target distribution is to generate B particles and resample them based on importance weights derived from scores exp(λr(y,l,P)), following the principle of importance sampling.

The sequential Monte Carlo (SMC) method [Del Moral et al., 2006] extends this concept to a time-sequential setting by maintaining B particles and updating their importance weights over time [Wu et al., 2023]. A straightforward weighting scheme involves evaluating the current batch of particles ${\left\{l_{i,\tau }\right\}}_{i=1}^{B}$ at time τ, computing their scores ${\left\{{\hat{y}}_{i}=r\left(l_{i,\tau },P\right)\right\}}_{i=1}^{B}$, and obtaining resampling weights through softmax normalization: $w_{i}=\frac{\exp\left({\hat{y}}_{i}\right)}{\sum_{j}\exp\left({\hat{y}}_{j}\right)}$. Particles are then resampled from the discrete distribution ${\left\{l_{i}\right\}}_{i=1}^{B}\sim \mathrm{Multinomial}\left(B,w=\left(w_{1},w_{2},\ldots ,w_{B}\right)\right)$ with probability proportional to their weights, as done in Pilot [Cremer et al., 2024].

### 4.4 Quantum Mechanical Calculations on Protein-Ligand Complexes

To benchmark Flowr.root’s affinity prediction head, we perform quantum mechanical (QM) calculations on protein-ligand complexes and correlate the results with predicted affinities. Calculations were conducted using ULYSSES [Menezes and Popowicz, 2022], a general-purpose semi-empirical library offering multiple molecular Hamiltonians. We employ GFN2-xTB [Bannwarth et al., 2019] with ALPB aqueous solvation [Ehlert et al., 2021]. GFN2-xTB is a simplified density functional method that parametrizes interparticle interaction integrals against reference data, significantly accelerating calculations [Bannwarth et al., 2019]—a critical advantage for large atomic systems such as protein-ligand complexes. Additionally, GFN2-xTB provides a balanced treatment of electrostatic interactions and incorporates state-of-the-art dispersion corrections, enabling accurate descriptions of non-bonded complexes.

Benchmark calculations on protein complexes extracted from the Schrödinger FEP dataset already contained protonated protein structures. For these cases, Flowr.root-generated ligands had to be protonated using open babel [O’Boyle et al., 2011]. Calculations were run on the full complexes, water molecules were excluded and solvation was treated only implicitly, except when otherwise stated. In the case of the kinase selectivity test, structures were extracted directly from the PDB and processed with open babel. This includes protonation of the protein pockets and of all ligands, along with their predicted total charges.

## 5 Results

To comprehensively assess Flowr.root’s capabilities, we conduct a systematic evaluation across three core areas: structure generation, affinity prediction, and inference-time scaling via importance sampling.

First, we establish the improvements regarding the model changes that we incorporated into the Flowr.root model compared to the recently proposed Flowr model. We do so by first showing the expressiveness of the ligand decoder backbone for unconditional molecule generation in 3D on the well established Geom-Drugs dataset and also compare against other recently introduced, state-of-the-art models in this domain, namely EQGAT-diff, SemlaFlow, ADiT, Megalodon and FlowMol3.

Next, to benchmark Flowr.root for pocket-conditional 3D molecule generation, we use the widely adopted CrossDocked2020 dataset Francoeur et al. [2020], despite its prevalences [Cremer et al., 2025], and compare against recent state-of-the-art diffusion- and flow-based generative models, namely Pocket2Mol, DiffSBDD, TargetDiff, DrugFlow, PILOT and Flowr. For a more challenging and meaningful evaluation of generative SBDD models, we further evaluate Flowr.root on the recently proposed Spindr dataset and compare against Flowr and the diffusion-based Pilot model Cremer et al. [2024].

For affinity prediction, we assess performance on Spindr, the Schrodinger FEP+ benchmark, and the OpenFE IndustryBenchmark, demonstrating the model’s ability to accurately predict binding affinities across diverse experimental measurements (${\text{pIC}}_{50}$, ${\text{pK}}_{i}$, ${\text{pK}}_{d}$, ${\text{pEC}}_{50}$).

We further demonstrate Flowr.root’s practical utility through domain adaptation via fine-tuning on project-specific in-house data and validate the model’s joint structure-affinity predictions through case studies on kinase selectivity (CK2α/CLK3), ligand optimization (TYK2, ERα, BACE1), and quantum mechanical validation.

Collectively, these evaluations establish Flowr.root as a versatile and accurate tool for structure-based drug design, achieving state-of-the-art performance in generative modeling while enabling efficient property-guided optimization and robust affinity prediction.

### 5.1 Unconditional Molecule Generation: Geom-Drugs

We first evaluate Flowr.root in an unconditional setting on the challenging Geom-Drugs dataset. This dataset is commonly used to benchmark 3D molecular generation models. The results in Table 1 show that Flowr.root outperforms state-of-the-art models, including the diffusion-based models EQGAT-diff and Megalodon, and recently released flow matching-based FlowMol3, and others, in several key metrics such as chemical validity, geometric accuracy, and energetic stability.

Flowr.root achieves the second highest RDKit-validity of 98.5% and a PoseBusters-validity of 94.0%, surpassing all other models significantly including FlowMol3 (94.0% vs. 91.9%). These results indicate that Flowr.root generates molecules that are both chemically valid and geometrically plausible. It is worth noting that RDKit validity only measures whether or not a molecule can be processed by RDKit, while PoseBusters-validity is a comprehensive measure of 3D plausibility [Buttenschoen et al., 2024].

In terms of energetic stability, as measured by the median change in potential energy after GFN2-xTB minimization (relaxation energy, $\text{Δ}E_{relax}$), Flowr.root achieves a value of 3.65 kcal/mol, outperforming FlowMol3 (3.83 kcal/mol) and substantially outperforming most other baselines apart from the diffusion-based Megalodon model (3.17 kcal/mol). This suggests that the generated conformations are close to local energy minima, reflecting strong data distribution learning capabilities.

Importantly, Flowr.root achieves a median relaxation RMSD of 0.07 Å, which is significantly lower than all other models, including FlowMol3 (0.39 Å) and Megalodon (0.41 Å). This demonstrates that the generated conformers require minimal adjustment during quantum mechanical relaxation, highlighting the geometric precision of the model’s outputs especially important for the development of structure-aware models with protein- and/or pocket-contexts, where the geometrically accurate placement of ligand conformations is crucial.

### 5.2 Pocket-Conditional Ligand Generation: CrossDocked2020

Next, we evaluate the performance of Flowr.root on the widely adopted CrossDocked2020 dataset, which tests the ability of models to generate ligands that fit within known protein pockets. Table 2 shows that Flowr.root outperforms both the original Flowr model and other recent state-of-the-art models in terms of PoseBusters-validity, strain energy, and docking performance.

Flowr.root achieves a PoseBusters-validity of 0.97, surpassing all other models, including the test set reference (0.95) itself. This highlights Flowr.root’s ability to generate highly valid ligands across various protein pockets. In terms of strain energy, Flowr.root also outperforms all other models, achieving 67.13 kcal/mol. This is significantly lower than Flowr (87.83 kcal/mol) and the next best model Pilot (110.48 kcal/mol), indicating Flowr.root’s capabilities to generate low-strained binding poses, suggesting that in contrast to other models generated molecules are energetically favorable and close to their pocket-dependent conformations. Further, Flowr.root achieves the best AutoDock-Vina score of −7.76 kcal/mol, outperforming all baseline models. This indicates that Flowr.root is capable of effectively exploring protein-ligand interactions, and coupled with the lowest Wasserstein distances for bond angles and bond lengths, demonstrates that Flowr.root not only generates chemically valid molecules but also accurately captures the geometric distribution of the CrossDocked2020 dataset. Here, Flowr.root achieves the lowest values (0.91 and 0.22, respectively), closely matching the distributions observed in the test set.

Finally, Flowr.root maintains efficient inference times (15.43s per ligand), which are competitive with or better than most baselines, especially considering the substantial gains in quality. In summary, these results establish Flowr.root as the leading model for pocket-conditional 3D ligand generation, combining high validity, energetic realism, geometric accuracy, and computational efficiency.

### 5.3 Pocket-Conditional Ligand Generation: Spindr

Here, we evaluate Flowr.root’s ability to generate valid, physically plausible ligands closely following the underlying data distribution - but this time on the more challenging test set of the Spindr dataset. We compare the non-pretrained Flowr.root^base^ model with Flowr and the diffusion-based Pilot model. Table 3 reveals that the Flowr.root base model significantly outperforms both Flowr and Pilot across all metrics.

Flowr.root achieves a PoseBusters-validity of 0.97, nearing the test set reference (0.99). Additionally, the strain energy statistics significantly decreases to 56.12 kcal/mol, further demonstrating the model’s ability to generate chemically valid and energetically favorable ligands. Flowr.root also performs better in docking accuracy, with a mean Vina score of −7.12 kcal/mol.

### 5.4 Affinity prediction: Spindr

**Figure 3:**
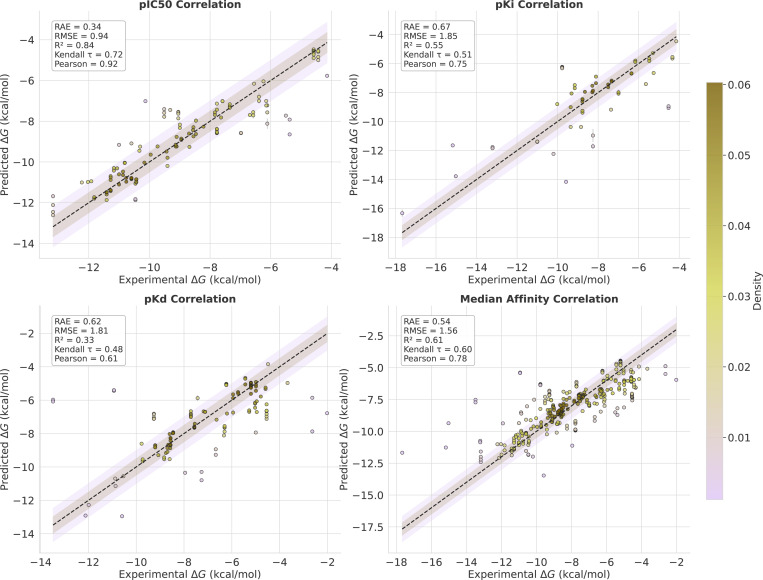
**Top left:** Correlation plot of Flowr.root-predicted ${\text{pIC}}_{50}$ in kcal/mol vs. experimental ${\text{pIC}}_{50}$ binding affinities across protein-ligand complexes on the Spindr test set, with shaded regions indicating 0.5 and 1 kcal/mol error boundaries, and color denoting density of predictions (the darker the denser). Error bars are reported as standard deviations from five seed runs. **Top right**: Correlation with experimental ${\text{pK}}_{i}$ affinities. **Bottom left** Correlation with experimental ${\text{pK}}_{d}$ affinities, and (**bottom right**) shows the correlation results if the median of all predicted affinities is used.

A central part of Flowr.root is its joint structure–affinity modeling. In Fig. 3 we provide a detailed analysis of affinity prediction performance on the Spindr test set. The affinity head of Flowr.root provides accurate predictions across various affinity types, including ${\text{pIC}}_{50}$, ${\text{pK}}_{i}$, and ${\text{pK}}_{d}$. For ${\text{pIC}}_{50}$, the model achieves a Pearson correlation of 0.92 and an R^2^ of 0.84, while for ${\text{pK}}_{i}$ and ${\text{pK}}_{d}$ we get Pearson correlations of 0.75 and 0.61, respectively. When aggregating predictions across all affinity types (median), the model achieves RAE 0.54, RMSE 1.56, R^2^ 0.61, τ 0.60, and Pearson 0.78. These results confirm that the affinity head, trained jointly with structure generation, provides accurate and reliable potency estimates with robust performance across different experimental affinity labels. Probing the Flowr.root^base^ model trained solely on Spindr without pre-training, we get significantly worse results with RAE 0.70, RMSE 1.95, R^2^ 0.39, τ 0.49, and Pearson 0.63 underlining the importance of the proposed large-scale pre-training pipeline. Note, in both cases we strictly follow the Plinder dataset split minimizing information leakage between train and test even when pre-training.

**Figure 4:**
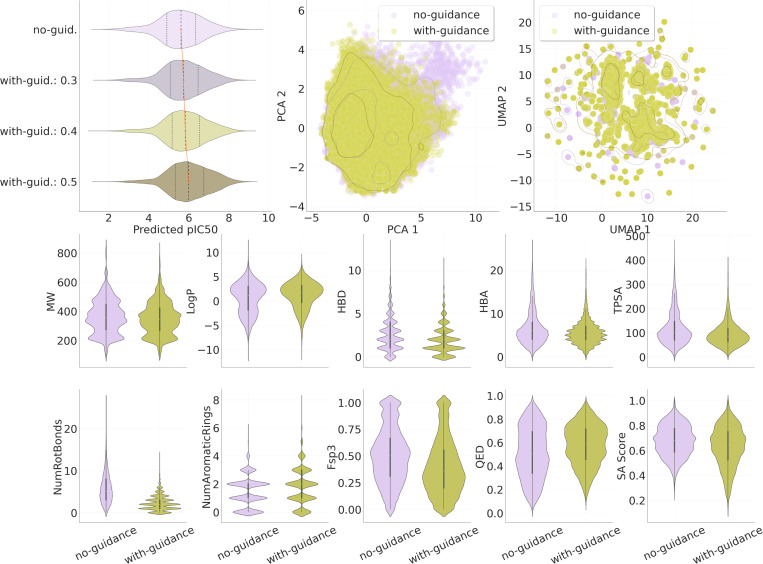
**Top left:** Inference-time steering via importance sampling on the Spindr test set using the Flowr.root model comparing the distribution of ${\text{pIC}}_{50}$ predictions of generated ligands across test set targets between un-guided, and mild to strongly guided steering. **Top right:** PCA and UMAP depiction of chemical space comparison between un-guided and strongly guided samples. **Bottom rows:** Distribution comparison between un-guided and strongly guided samples regarding different chemical properties, namely molecular weight (MW), logP, number of hydrogen donors (HBD) and acceptors (HBA), topological surface area (TPSA), number of rotatable bonds (NumRotBonds) and aromatic rings (NumAromaticRings), fraction of $sp^{3}$ carbons (Fsp3), druglikeness (QED) and synthesizability (SA Score).

Beyond predictive accuracy, Flowr.root enables property-guided generation via inference-time steering. Fig. 4 illustrates the effect of importance sampling-based steering on the predicted affinity distribution of generated ligands on the Spindr test set. Without guidance, the mean predicted ${\text{pIC}}_{50}$ is 5.60 (std 1.11). As the steering duration increases (from 0.3 to 0.5 of the trajectory), the mean predicted ${\text{pIC}}_{50}$ shifts progressively to higher values (5.75, 5.84, 6.02), while the standard deviation remains stable. This demonstrates that the model can be effectively biased toward predicted higher-affinity ligands during generation, without sacrificing chemical or geometric validity, as can be seen in the bottom row of Fig. 4, where we show the distribution of important chemical properties comparing un-guided with guided (0.5 steering duration) samples across targets on the Spindr test set. Interestingly, we find that guidance consolidates the sample space, leading to mostly more compact distributions. This can also be seen in the chemical space comparison on the left of the top row of Fig. 4, where we overlay the chemical spaces of the un-guided and guided samples using both PCA and UMAP on molecular fingerprints.

### 5.5 Affinity prediction: Schrodinger FEP+ and OpenFE

Here, we evaluate the performance of the affinity prediction module of Flowr.root through several case studies using widely recognized open source benchmarks. We begin by assessing the model’s ability to predict binding affinities using the Schrodinger FEP+ and OpenFE IndustryBenchmark datasets, respectively, comparing Flowr.root to state-of-the-art methods. These benchmarks offer a robust test of the model’s prediction capabilities across various targets and affinity types.

#### Schrödinger FEP+ Benchmark

**Figure 5:**
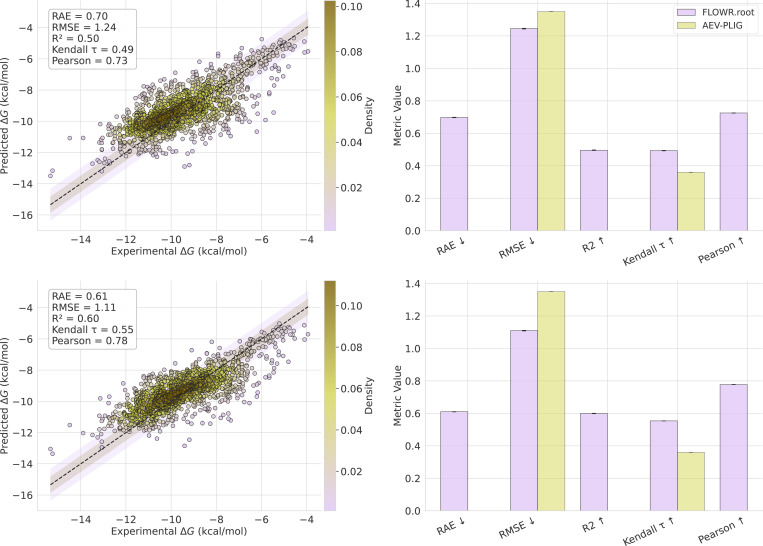
**Top left:** Correlation plot of Flowr.root-predicted ${\text{pK}}_{d}$ in kcal/mol vs. experimental binding affinities across protein-ligand complexes of the Schrodinger FEP+ benchmark dataset, with shaded regions indicating 0.5 and 1 kcal/mol error boundaries, and color denoting density of predictions (darker means more dense). Error bars are reported as standard deviations from five seed runs. **Top right:** Mean values of different correlation metrics with error bars indicating the 95% confidence interval over the five different seed runs. **Bottom left:** Correlation plot of Flowr.root-predicted binding affinities as mean over ${\text{pK}}_{d}$, ${\text{pK}}_{i}$, and ${\text{pIC}}_{50}$ in kcal/mol, and respective correlation mean values. **Bottom right:** Correlation statistics of the combined prediction for all affinity types.

To evaluate the affinity prediction performance of Flowr.root on a challenging and high-fidelty dataset, we use the Schrodinger FEP+ benchmark, which provides experimentally determined binding affinities for protein-ligand complexes. The results are presented in Figure 5, where we show the correlation of Flowr.root-predicted binding affinities against the experimental values.

The scatter plot in the top left panel of Figure 5 illustrates the correlation between Flowr.root-predicted ${\text{pK}}_{d}$ values and experimental binding affinities, with shaded regions indicating error bounds of 0.5 and 1.0 kcal/mol, respectively. The denser regions of the plot (colored darker) represent higher prediction frequencies, indicating where the model performs best. The top right panel shows the mean values of various correlation metrics, with error bars representing the 95% confidence interval across five seed runs comparing Flowr.root with AEV-PLIG. Additionally, the bottom left panel shows a combined correlation plot across multiple affinity types (median of ${\text{pK}}_{d}$, ${\text{pK}}_{i}$, and pIC50), emphasizing Flowr.root’s ability to predict diverse affinity types that can also be combined to get more robust predictions.

Flowr.root demonstrates strong performance with an RAE of 0.610 and an RMSE of 1.11 kcal/mol. The R-squared $\left(R^{2}\right)$ value is 0.60, and the Kendall τ coefficient is 0.55, indicating a high degree of correlation between the predicted and experimental values. These results are notably superior to the baseline model, AEV-PLIG, which achieves an RMSE of 1.35 and a Kendall τ of 0.36, highlighting the advantage of Flowr.root in affinity prediction. Since this benchmark dataset comprises protein-ligand complexes with a wide variety of experimental validation, it is not surprising that combining the different affinity outputs of Flowr.root results in a more robust performance.

#### OpenFE IndustryBenchmark

**Figure 6:**
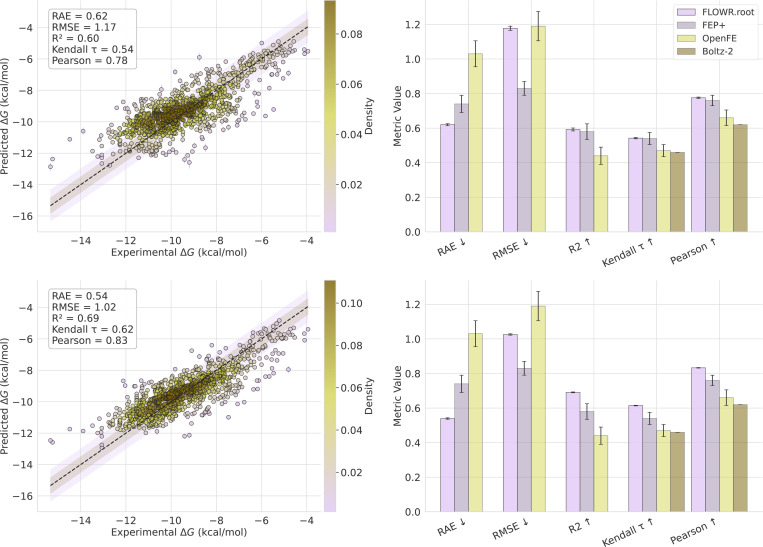
**Top left:** Correlation plot of Flowr.root-predicted ${\text{pK}}_{d}$ in kcal/mol vs. experimental binding affinities across protein-ligand complexes of the OpenFE INDUSTRYbenchmark dataset, with shaded regions indicating 0.5 and 1 kcal/mol error boundaries, and color denoting density of predictions (the darker the more dense). Error bars are reported as standard deviations from five seed runs. **Top right:** Mean values of different correlation metrics with error bars indicating the 95% confidence interval over the five different seed runs. **Bottom left:** Correlation plot of Flowr.root-predicted binding affinities as median over ${\text{pK}}_{d}$, ${\text{pK}}_{i}$ and ${\text{pIC}}_{50}$ in kcal/mol and respective correlation mean values. **Bottom right:** Correlation statistics of the combined prediction for all affinity types.

We further validate Flowr.root on the OpenFE IndustryBenchmark dataset, which is a curated subset of the Schrodinger FEP+ dataset ^1 2^. The top left panel of Figure 6 presents the scatter plot for Flowr.root-predicted ${\text{pK}}_{d}$ values versus experimental binding affinities. As with the Schrodinger FEP+ benchmark, the plot clearly shows a strong correlation with minimal deviation, suggesting that Flowr.root can predict binding affinities across a broad range of protein-ligand complexes. In the top right panel we show the performance of Flowr.root using various error and correlation metrics in comparison to OpenFE, FEP+, AEV-PLIG and Boltz-2. In the bottom left panel, we show the combined affinity prediction (median over ${\text{pK}}_{d}$, ${\text{pK}}_{i}$, and ${\text{pIC}}_{50}$).

Flowr.root achieves an RAE of 0.54 and an RMSE of 1.02 kcal/mol, with a Pearson correlation of 0.83 and a Kendall τ of 0.62. These results surpass those of OpenFE (RAE = 1.03, RMSE = 1.19, Kendall τ=0.47, Pearson = 0.66) and FEP+ (RAE = 0.74, RMSE = 0.83, Kendall τ=0.54, Pearson = 0.76).

The results demonstrate that Flowr.root outperforms physics-based models, namely OpenFE and FEP+, on almost all error and correlation metrics and significantly outperforms recent state-of-the-art ML-based models AEV-PLIG and Boltz-2 across all metrics. Notably, Flowr.root achieves this while being 3x faster than AEV-PLIG, 200x faster than Boltz-2 (which in contrast only requires protein sequence and ligand SMILES string), and over 10000x faster than OpenFE and FEP+. However, we also want to emphasize that the Schrodinger FEP+ dataset (and with that the OpenFE dataset) is likely significantly overlapping with the training data in terms of ligand and target space. While this applies to AEV-PLIG and Boltz-2 as well allowing for a somewhat fair comparison, we do not expect these results to indicate significant generalization capabilities.

### 5.6 Domain Adapation via Finetuning: In-House Data

Here we investigate the performance of Flowr.root on in-house project-specific data. As expected, the model exhibits no generalization to the presented chemical and bioactivity space, resulting in negligible correlation between predicted and experimental binding affinities (RMSE: 2.65, $\left(R^{2}\right)$: −2.18, Kendall τ=0.24, and Pearson: 0.39). This limitation extends to other state-of-the-art models, including Boltz-2, highlighting a fundamental challenge in the field.

However, Flowr.root distinguishes itself through its capacity for straightforward yet effective fine-tuning on project-specific data. This enables a critical shift toward the underlying data distribution, facilitating better understanding of the structure-activity landscape.

We fine-tuned the model using parameter-freezing, yielding ∼ 10M trainable parameters trained over 50 epochs. Since our in-house dataset comprises ${\text{pIC}}_{50}$-annotated datapoints, only the ${\text{pIC}}_{50}$-head and alongside the backbone were fine-tuned.

**Figure 7:**
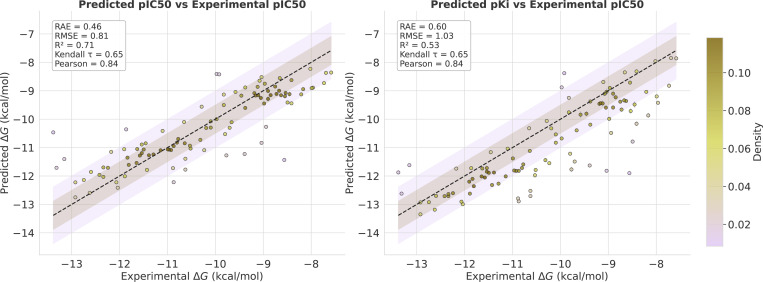
Evaluation of Flowr.root’s ${\text{pIC}}_{50}$ and ${\text{pK}}_{i}$ affinity prediction accuracy on a challenging in-house project-specific dataset comprising around 1,000 experimentally tested ligands after finetuning. We examine the performance of Flowr.root on a hold-out test set generated by a random split.

As can be seen in Fig. 7 (left), fine-tuning yielded substantial performance improvements: RMSE of 0.81, $R^{2}$ of 0.71, Kendall τ of 0.65, and Pearson correlation of 0.84. Notably, $\text{p}K_{i}$ prediction also improved significantly despite not being explicitly optimized (Fig. 7, right).

These results underscore a critical observation: While covering chemical space through ligand generation presents manageable challenges, generalizing bioactivity landscapes represents an entirely different—and significantly more complex—problem. However, mode shifts via fine-tuning seem to provide the necessary pathway to address this complexity. Thus, rather than expecting models to ”train once and work universally”, we must embrace continuous refinement as an integral component of the discovery process, recognizing that model utility grows through sustained interaction with project-specific data and interpreting generative models rather as companions than standalone tools.

### 5.7 Case studies

To demonstrate the practical utility and versatility of Flowr.root for structure-based drug design, we present three case studies that systematically evaluate different aspects of the framework’s capabilities. First, we investigate multi-objective optimization through a kinase selectivity study, where we simultaneously maximize binding affinity for the on-target kinase CK2α while minimizing off-target activity against CLK3. This case study demonstrates how Flowr.root can address one of the most challenging aspects of kinase drug discovery—achieving selectivity among structurally homologous ATP-binding sites.

Second, we benchmark the framework’s conditional ligand generation performance against quantum mechanical binding energy calculations using TYK2 kinase, ERα and BACE1 as model systems. These validation studies assess the correlation between Flowr.root-predicted binding affinities and computationally demanding QM binding energies, while providing mechanistic insights into the structural features that govern binding affinity in the generated ligands.

### 5.8 Ligand Selectivity: CK2α and CLK3

The human kinome consists of over 500 kinases [Manning et al., 2002] that share conserved catalytic domain structures, particularly in their ATP-binding sites, where most competitive inhibitors bind. This structural homology, while functionally important for cellular signaling pathways, presents a major challenge for kinase selectivity—the ability of small molecule inhibitors to achieve potent inhibition of specific kinase targets while avoiding off-target interactions with structurally similar kinases. To demonstrate the capability of our framework for multi-objective optimization, we perform a selectivity study targeting selectivity between CK2α (on-target, PDB: 3pe1) and CLK3 (off-target, PDB: 6khf).

We compare two optimization strategies using importance sampling: joint optimization that simultaneously maximizes the predicted binding affinity on CK2α while minimizing CLK3 affinity formulated as dual-objective, versus the single optimization which only maximizes CK2α. We generated 1,000 ligands for each set using the protein pockets from PDB 3pe1 [Battistutta et al., 2011] and 6khf [Lee et al., 2019] after rotational alignment.

**Figure 8:**
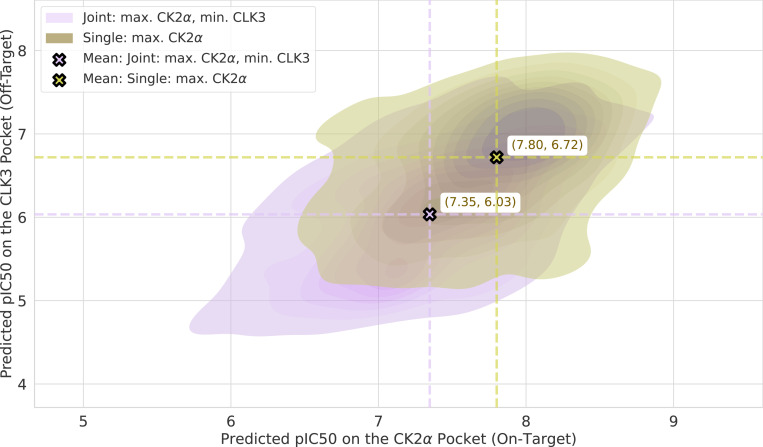
Kernel density estimation plot comparing joint optimization (purple, maximizing CK2α while minimizing CLK3) versus single optimization (green, maximizing CK2α only) on predicted pIC50 values. Black X markers show mean values with dashed reference lines. Joint optimization achieves improved selectivity with lower off-target CLK3 activity (mean pIC50 = 6.03 vs. 6.72) while maintaining comparable on-target CK2α potency, demonstrating kinase selectivity optimization.

Our results show that joint optimization successfully achieves improved selectivity profiles compared to single-target optimization. The joint approach generated ligands with significantly lower off-target CLK3 activity (mean pIC50 = 6.03) compared to single optimization (mean pIC50 = 6.72), while maintaining comparable on-target CK2α potency as shown in the distribution plots in Figure 8 with mean pIC50 of 7.35 for the joint set on CK2α.

**Figure 9:**
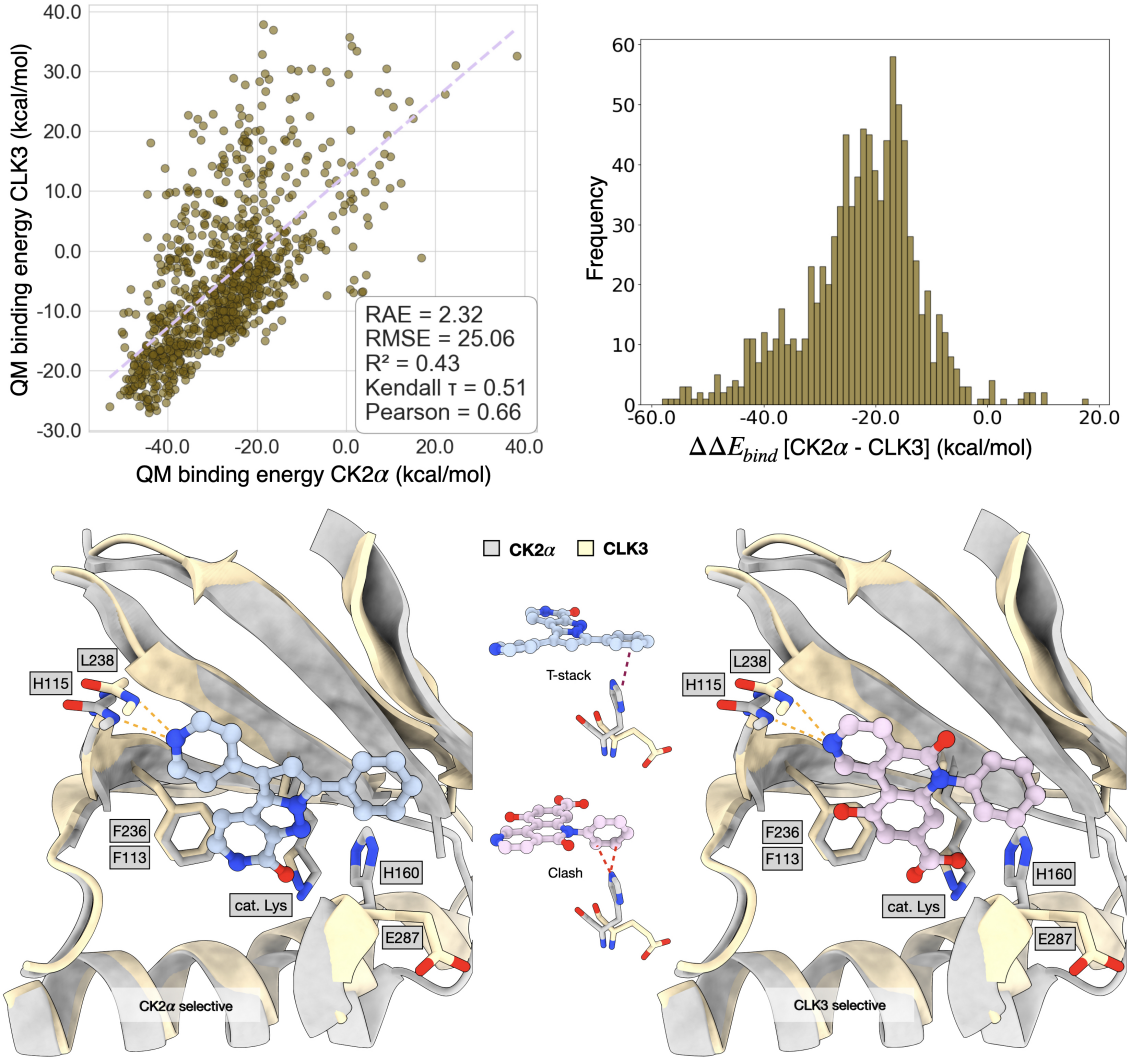
Quantum mechanical analysis of the joint ligand design to achieve selectivity towards CK2α, while minimizing binding to CLK3. **Top left:** Quantum mechanical-calculated binding energy for CLK3 vs. CK2α. Ligands for which the binding energy is higher than 40.0 kcal/mol were excluded from the plot (≈ 5 %). The plot shows systematically lower binding energies for CK2α, indicating selectivity. **Top right:** the distribution of the difference in binding energies for all investigated ligands. The skewed distribution peaks at about −17.0 kcal/mol. **Lower left:** example of a CK2α-selective ligand and its interaction patterns with the kinases’ pockets. **Lower Right:** example of a CLK3-selective ligand and its interaction patterns with the kinases’ pockets. **Lower Middle:** interactions of the two ligands with the residue H160.

We further benchmark our approach using an alternative protocol. To this end, we took the 1,000 Flowr.root-generated ligands from the joint optimization and performed QM binding energy calculations. We excluded all complexes for which CLK3 binding energies were above 40.0 kcal/mol, as these resulted from structural clashes (Å 5.6 % of all complexes). Fig. 9 (upper-left block) shows a scatter plot of CK2α against CLK3 binding energies. The scatter shows a linearized correlation shifted towards lower CK2α binding energies, corroborating the success of the approach. Further, we record several outliers from linearity, which result from the fact that Flowr.root tries to further penalize ligands in the CLK3 pocket. In this task, however, the most relevant metric is the relative binding energy of a given ligand towards the pockets of each kinase. Fig. 9 (upper-right block) shows the distribution of relative binding energies in these two kinases. The distribution, peaked at about −17.0 kcal/mol, is skewed, with a quick decay towards higher relative binding energies (less selective) and a slower decay on lower relative binding energies (more selective) further validating our approach.

In this task, it is particularly interesting to analyze ligands that minimize and maximize the relative binding energies between the two kinases. The lower blocks of Fig. 9 compare a CK2α-selective $\left(\text{ΔΔ}E_{bind}=-58.18\;\text{kcal}/\text{mol}\right)$ against a CLK3 binder $\left(\text{ΔΔ}E_{bind}=+18.00\;\text{kcal}/\text{mol}\right)$, indicating the mechanisms, with which Flowr.root tried to accomplish selectivity. Two major protein-ligand interactions were explored. On one hand, the interaction with the kinases’ hinge. In both cases, the hydrogen bond between the generated ligand and L238 of CLK3 shows a suboptimal angular orientation. Conversely, the CK2α-selective binder shows a quasi-optimal hydrogen bond orientation with CK2α’s H115, with the angle $H-{\hat{N}}_{ar}-C_{ar}$ reaching a value of 114.2°. In the case of the CLK3-selective binder, the equivalent angle with CK2α’s main chain H of H115 goes up to 137.9°. Additional weakening of the hydrogen bond to CK2α’s hinge is achieved by increasing the $H-\hat{N}$ distance, which goes from 2.77 Å in the CK2α-selective binder to 2.97 Å in the CLK3-selective one.

The second mechanism is related to interactions with H160. In previous work [Grygier et al., 2023], we identified H160 as a critical residue conferring sub-nM affinity of the inhibitor silmitasertib to CK2α. Here too, we observe that Flowr.root explores interactions with this residue to achieve selectivity. On the CK2α-selective binder, Flowr.root orients the ligand’s phenyl substituent to optimize a T-stack interaction. On the CLK3-selective binder, a similar substituent is rotated to yield a short, repulsive contact. This is possible because the side chain of E287, the residue in a similar position in CLK3, points in another direction.

We conclude that, in order to achieve selectivity between two similar kinases, Flowr.root exploited two previously identified elements of selectivity. This concerns the hinge region of kinases, where better binders show shorter hydrogen bond distances and more adequate angles. Simultaneously, Flowr.root also explored lipophilic interactions with the two kinases, optimizing a T-stack with a residue critical for the high potency of drugs currently in clinical trials. We stress that none of these interactions could have been passed to the model during training or in guiding selectivity.

### 5.9 TYK2, ERα, and BACE1

As shown in previous sections, Flowr.root generates novel chemical matter conditioned on a protein pocket. The generated molecules are chemically reasonable and are not heavily strained, unlike previous generative models. Additionally, the predicted affinity data reliably reproduces experimental distributions. In this section, we benchmark how the whole Flowr.root workflow behaves on the task of conditional ligand generation. To this end, we chose the TYK2 kinase, ERα, and BACE1 as targets from the Schrodinger FEP+ dataset.

Testing whether the Flowr.root-generated ligands are good binders requires effective metrics. In this case, we chose to benchmark the Flowr.root poses using QM binding energy analysis. QM calculations on protein-ligand complexes offer scoring functions; therefore we aimed at finding a correlation between QM binding energies and the median of predicted binding affinities.

**Figure 10:**
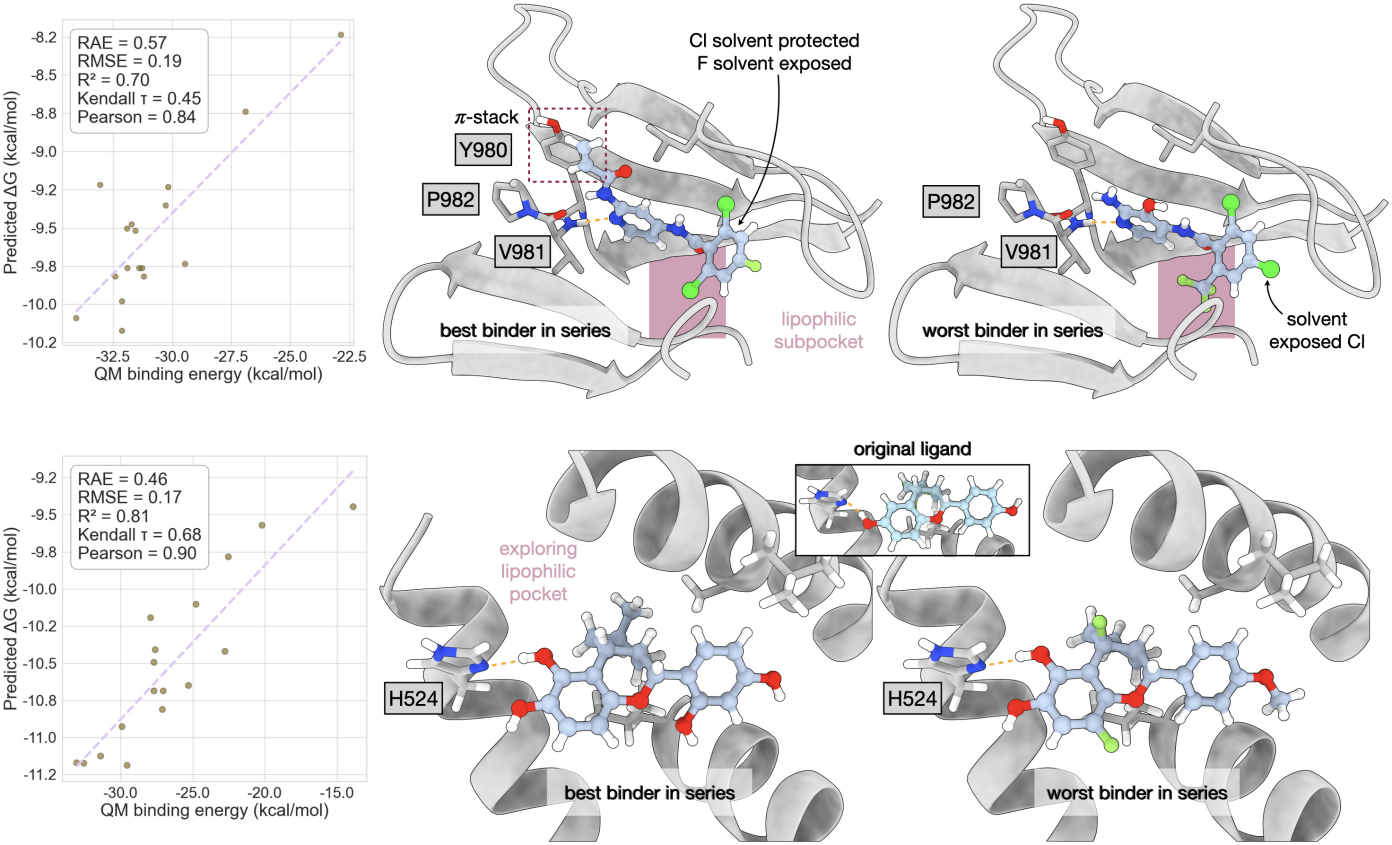
Flowr.root binding affinity validation using quantum mechanical calculations. Benchmark cases of the TYK2 kinase (top block) and ERα (lower block). **Top Left:** correlation plot between Flowr.root predicted affinities and the quantum mechanical binding energies for TYK2. **Top Middle:** example of the best binder in the series and its interactions with the pocket. **Top Right:** example of the worst binder in the series and its interactions with the pocket. **Bottom Left:** correlation plot between Flowr.root predicted affinities and the quantum mechanical binding energies for ERα. **Bottom Middle:** example of the best binder in the series and its interactions with the pocket. **Bottom Right:** example of the worst binder in the series and its interactions with the pocket.

We first look at the example of the TYK2 kinase (Fig. 10, upper block). We find a good correlation between the QM-calculated binding energies and the Flowr.root-predicted median affinities, with an R^2^ of 0.70, a Pearson correlation coefficient of 0.84, a Kendall τ of 0.45, RMSE of 0.19, and RAE of 0.57. From a chemical viewpoint, it is more interesting to compare the structures of the best and worst Flowr.root-predicted ligands. We see that, despite some common features, the model generalizes effectively the hinge-binding motif, and the solvent-exposed residue of the ligand. Importantly, all ligands retained the hydrogen bond acceptor to V981, a critical hinge residue already in the original ligand structure. Binding to a kinase’s hinge is very important in kinase inhibition, kept by Flowr.root-generated chemical matter. Interestingly, no ligand improved the hydrogen-bond geometry with P982, despite some variations in the chemotypes exhibited by the hinge-binding heads. The major differences take place in how specific interactions are exploited. Particularly critical for the best binder in this series is the π-stack interaction established with Y980. But also the fact that this ligand protects the least lipophilic halogens from solvent exposure. We also calculated ligand deformation energies, which for this series lie between 19.32 kcal/mol and 44.29 kcal/mol.

A similar correlation was obtained for ERα (Fig. 10, lower block), however, in this case, all the statistical metrics improved: The R^2^ is 0.81, the Pearson correlation coefficient is 0.90, the Kendall τ is 0.68, RMSE is 0.17 and RAE 0.46. Some variability is expected between systems, and in the case of ERα we observe less outliers than in the TYK2 example. In the case of ERα, we observed that Flowr.root retained most of the ligand’s structure, improving mostly the interactions with the residue H524. In the structure of the original ligand there is a poorly-oriented hydrogen bond with H524, which is effectively corrected even in the case of the worst binder: the Flowr.root-generated ligands exhibited better geometries for this hydrogen bond. Similar to the case of the TYK2 kinase, we calculated ligand deformation energies, which were between 24.36 kcal/mol and 38.30 kcal/mol. These values are significantly lower than those of Tables 2 and 3, which indicate that the values of 67.13 kcal/mol and 56.12 kcal/mol might be upper boundaries.

Finally, we also analysed a benchmark on BACE1, where we obtained the best correlation of this series between Flowr.root-predicted affinities and the QM calculated ones. This is seen in the lowest RAE and RMSE coupled with the highest R^2^, Pearson correlation coefficient, and Kendall τ of this series of benchmarks. We see that in all cases the ligands retain a water-mediated hydrogen bond with F108, which results in high affinities overall. The major differences are in the nature of the nitrogen-containing group near Y71, which seems to favour more lipophilic groups, but also the substituents in the aromatic ring T-stacking the residue W115. We note that the T-stack interaction is favoured by electron-rich phenyl rings, which is not the case of the worst binder in the series. This seems then to be one of the major effects controlling affinity to BACE1.

## 6 Conclusion

In this work, we introduced Flowr.root, a foundational framework for structure-based generative chemistry that unifies multi-mode ligand generation with binding affinity prediction and domain adaptation within a single E(3)-equivariant flow matching architecture. The framework supports *de novo* design, interaction-guided generation, and fragment-based elaboration, enabling applications spanning from early-stage hit identification to late-stage lead optimization. By coupling structural prediction with dedicated affinity and confidence heads, Flowr.root directly integrates potency estimation into the generative trajectory, allowing property-guided molecule design through inference-time steering. Our three-stage training paradigm—large-scale pretraining on billions of ligands and millions of mixed-fidelity protein-ligand complexes, refinement on curated high-fidelity datasets, and project-specific adaptation—enables the model to capture broad chemical diversity while remaining efficiently adaptable to specialized structure-activity landscapes.

Our comprehensive evaluation demonstrates that Flowr.root advances the state of the art across multiple dimensions spanning from unconditional molecule to structure-aware ligand generation consistently outperforming recent diffusion- and flow-based approaches. For affinity prediction, Flowr.root achieves robust correlation with experimental measurements across diverse assay types, matching or exceeding the performance of computationally expensive physics-based and clearly outperforming ML-based methods while operating orders of magnitude faster. Critically, the model predictions maintain reliability across both public benchmarks and project-specific datasets when followed by targeted fine-tuning, demonstrating practical utility in real-world medicinal chemistry workflows.

Quantum mechanical validation studies on TYK2 kinase, ERα, and BACE1 provide evidence of Flowr.root’s capacity to generate chemically meaningful ligands and binding poses. Strong correlations between model-predicted affinities and rigorous QM binding energies—coupled with mechanistic analysis revealing that generated ligands systematically exploit key structural motifs such as hinge-binding interactions, hydrogen bond geometries, and aromatic stacking—demonstrate that the model has internalized fundamental principles of protein-ligand interactions. Our kinase selectivity study between CK2α and CLK3 further illustrates that through affinity-guided generation, Flowr.root can help finding selectivity mechanisms. Overall, Flowr.root provides a concrete step toward the vision of establishing continuously evolving frameworks that refine their understanding through sustained interaction with project-specific data, offering a foundational yet adaptable architecture that generalizes broadly, and adapts flexibly to specialized domains integrating well into medicinal chemistry workflows.

Nevertheless, important limitations remain. The reliance on public datasets, despite extensive curation, introduces biases from noisy affinity measurements, overrepresented protein families, and uneven chemical diversity. While higher-fidelity datasets partially mitigate these issues, their limited scale constrains model calibration, particularly for underexplored targets. Project-specific adaptation, though powerful, requires carefully chosen objectives and sufficient assay data; otherwise, models risk overfitting to narrow distributions. Additionally, Flowr.root requires the binding pocket to be known and preferably in a holo conformation, leaving the challenge of modeling protein pocket flexibility to future work. Finally, the predictions of Flowr.root remain approximations that cannot substitute for experimental validation.

Looking forward, several directions appear promising. Expanding project-level adaptation to include reinforcement learning or active learning frameworks with both physics-based and experimental feedback may enable continuous refinement during discovery campaigns. Further, enhancing synthetic accessibility estimation and coupling to reaction-based generative models would improve downstream feasibility, ensuring that generated molecules are not only potent but also synthetically accessible.

## Figures and Tables

**Figure 2:**
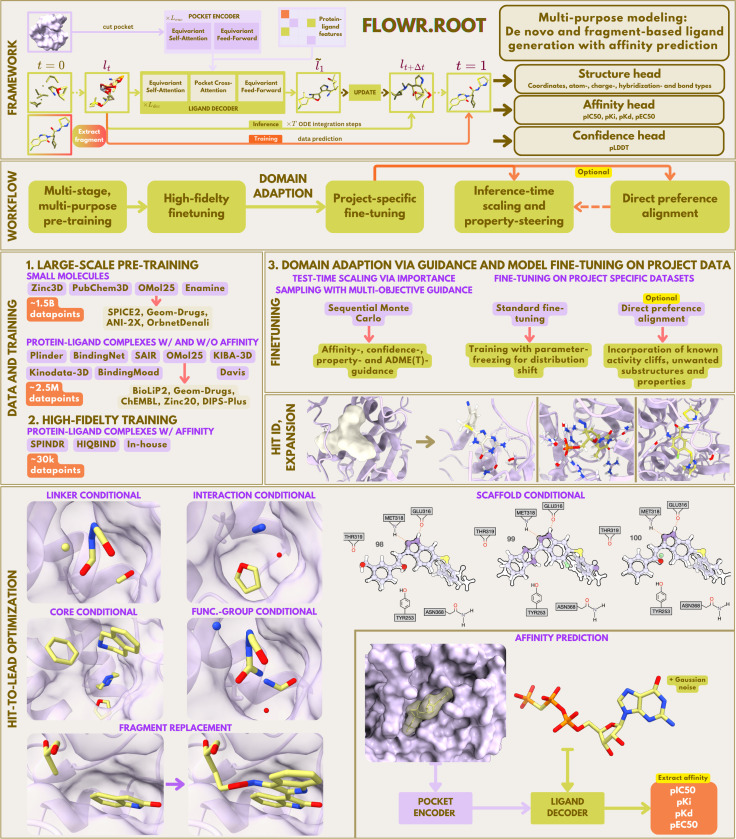
Graphical overview of the Flowr.root framework. Flowr.root is a flow matching-based framework for joint prediction of 3D ligand structure, binding affinity, and confidence estimation. The model follows a multi-stage training paradigm: large-scale pre-training on small molecules and mixed-fidelity protein-ligand complexes, followed by high-fidelity dataset training, with optional project-specific domain adaptation. Domain adaptation is enabled through standard or LoRA-based fine-tuning, direct preference alignment, and inference-time scaling via importance sampling with multi-objective guidance. The framework supports flexible conditional generation modes including scaffold-, linker-, core-, interaction-, and functional-group-conditional generation, as well as fragment or custom substructure replacement.

**Figure 11:**
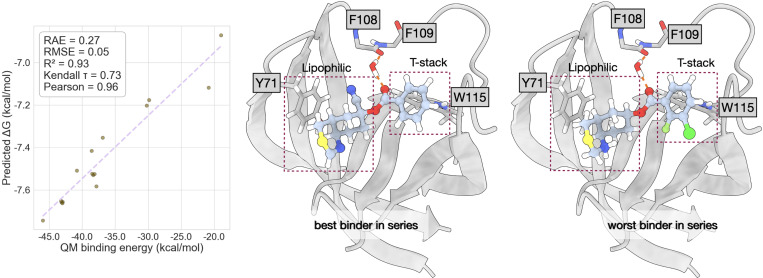
Flowr.root binding affinity validation using quantum mechanical calculations. Benchmark case of BACE1. **Left:** correlation plot between Flowr.root predicted affinities and the respective quantum mechanical binding energies. **Middle:** example of the best binder in the series and its interactions with the pocket. **Right:** example of the worst binder in the series and its interactions with the pocket.

**Table 1:** Evaluation and comparison of Flowr.root unconditional base model on Geom-Drugs. Benchmark comparison of the non-pretrained Flowr.root ligand-only base model against EQGAT-diff, ADiT, SemlaFlow, Megalodon, FlowMol3 on the Geom-Drugs dataset. We follow the conventions in this field and sample 10,000 molecules with molecule sizes randomly sampled form the test set. We evaluate the performance of Flowr.root on RDKit- and PoseBusters-validity, the change in potential energy resulting from GFN2-xTB minimization $\left(\text{Δ}E_{relax}\right)$ and the all-atom RMSD between the predicted and GFN2-xTB minimized conformations (Relax RMSD). All values denote the sample mean with the 95% confidence interval over five replicate runs with different seeds as subscripts. For $\text{Δ}E_{relax}$ and Relax RMSD we report the median.

Model	RDKit-valid (%) ↑	PB-valid (%) ↑	$\text{Δ}E_{relax}↓$	Relax RMSD ↓	Params (M)
EQGAT-diff	86.0 _± 0.9_	77.6 _± 0.8_	6.51 _± 0.16_	0.60 _± 0.01_	12
ADiT	99.9 _± 0.0_	82.7 _± 0.8_	79.32 _± 1.00_	1.30 _± 0.02_	150
SemlaFlow	95.5 _± 0.5_	88.5 _± 1.3_	31.9 _± 2.30_	0.24 _± 0.03_	40
Megalodon	94.8 _± 0.3_	86.6 _± 0.7_	3.17 _± 0.11_	0.41 _± 0.01_	60
FlowMol3	99.9 _± 0.1_	91.9 _± 0.7_	3.83 _± 0.08_	0.39 _± 0.01_	6

${\text{FLOWR.ROOT}}_{\text{UNCOND.}}^{\text{BASE}}$	98.5 _± 0.2_	94.0 _± 0.2_	3.65 _± 0.07_	0.07 _± 0.02_	34

Train set	100.0 _± 0.0_	93.2 _± 0.1_	-	-	

**Table 2:** Evaluation and comparison of Flowr.root base model on CrossDocked2020. Benchmark comparison of the the non-pretrained Flowr.root base model against Pocket2Mol, TargetDiff, DiffSBDD, Pilot, DrugFlow and Flowr on the CrossDocked2020 test dataset. We follow the conventions in this field and sample 100 ligands per test target, of which there are 100. We evaluate the most expressive metrics, namely PoseBusters-validity, GenBench3D strain energy, AutoDock-Vina scores and the Wasserstein distance of the generated ligands’ bond angles (BondA.W1) and bond lengths (BondL.W1) distributions relative to the test set. For all values, we report the mean across ligands and targets and the average standard deviation across targets as subscripts. For all models, we ran all evaluations on the subset of RDKit-valid ligands.

Model	PB-valid ↑	Strain↑	Vina score↓	Vina score^MIN^↓	BondA.W1↓	BondL.W1[10^−2^]↓	Size	Time (s) ↓
Pocket2Mol	0.76 _± 0.39_	147.22 _± 61.41_	−4.72 _± 1.47_	−5.80 _± 1.26_	2.04	0.66	17.04 _± 4.11_	2320 _± 45_
DiffSBDD	0.38 _± 0.46_	519.03 _± 251.32_	−2.97 _± 5.21_	−4.71 _± 3.30_	7.00	0.51	24.85 _± 8.94_	160.31 _± 73.30_
TargetDiff	0.57 _± 0.46_	294.89 _± 136.32_	−5.20 _± 1.79_	−5.82 _± 1.60_	7.76	0.42	22.79 _± 9.46_	3228 _± 121_
DrugFlow	0.75 _± 0.39_	120.21 _± 73.28_	−5.66 _± 1.78_	−6.10 _± 1.62_	2.11	0.38	21.14 _± 6.81_	-
Pilot	0.83 _± 0.33_	110.48 _± 87.47_	−5.73 _± 1.72_	−6.21 _± 1.65_	1.75	0.33	22.58 _± 9.77_	295.42 _± 117.35_

Flowr	0.92 _± 0.22_	87.83 _± 74.30_	−6.29 _± 1.56_	−6.48 _± 1.45_	0.96	0.27	22.28 _± 9.78_	12.05 _± 8.01_
Flowr.root^BASE^	0.97 _± 0.22_	67.13 _± 53.05_	−7.76 _± 0.55_	−7.93 _± 0.42_	0.91	0.22	22.41 _± 8.95_	15.43 _± 6.22_

Test set	0.95 _± 0.21_	75.62 _± 57.29_	−6.44 _± 2.74_	−6.46 _± 2.61_	-	-	22.75 _± 9.90_	-

**Table 3:** Evaluation and comparison of Flowr.root with Pilot and Flowr on Spindr. Benchmark comparison of the non-pretrained Flowr.root^base^ model against Flowr and the diffusion-based Pilot model on the Spindr test dataset. We sample 100 ligands per target across models. The evaluation includes PoseBusters-validity (PB-valid), strain energy calculated using GenBench3D, and AutoDock-Vina scores (kcal/mol). Additionally, we report the Wasserstein distance of the generated ligands’ bond angles (BondA.W1), bond lengths (BondL.W1) and dihedral angles (DihedralW1) distributions relative to those in the Spindr test set. Ligand sizes for all models are sampled uniformly with a −10%/+10% margin around the respective reference ligand size. All values are mean values across ligands and targets and we denote standard deviations across targets as subscripts.

Model	PB-valid ↑	Strain energy ↓	Vina score ↓	Vina score^MIN^ ↓	BondA.W1 ↓	BondL.W1 [10^−2^] ↓	DihedralW1 ↓

Pilot	0.79 _± 0.21_	120.10 _± 71.61_	−6.30 _± 0.96_	−6.68 _± 1.07_	1.82	0.42	5.52

Flowr	0.93 _± 0.22_	90.05 _± 52.18_	−6.93 _± 0.92_	−7.22 _± 0.92_	1.08	0.35	3.88
Flowr.root^BASE^	0.97 _± 0.10_	56. 12 _± 37.81_	−7.12 _± 0.76_	−7.36 _± 0.76_	0.60	0.41	3.56

Test set	0.99 _± 0.04_	43.27 _± 41.85_	−7.69 _± 2.00_	−7.88 _± 2.00_	-	-	-

**Figure 12:**
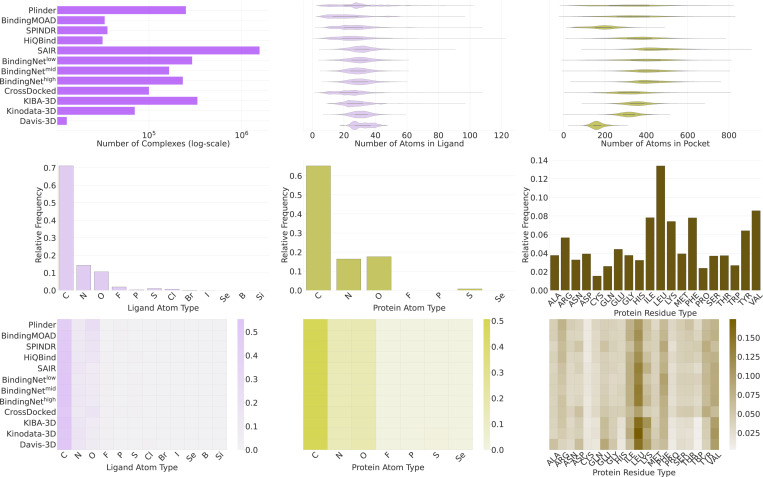
Distributions of ligand atom types, protein atom types, and protein residue types across multiple datasets. **Top:** Normalized frequencies of each type aggregated over all datasets. **Bottom:** Per-dataset distributions visualized as heatmaps (logarithmic scale for atom types), highlighting differences in composition between datasets. Only the 20 standard amino acids are shown for residue types; hydrogens are excluded from atom type analyses.

**Figure 13:**
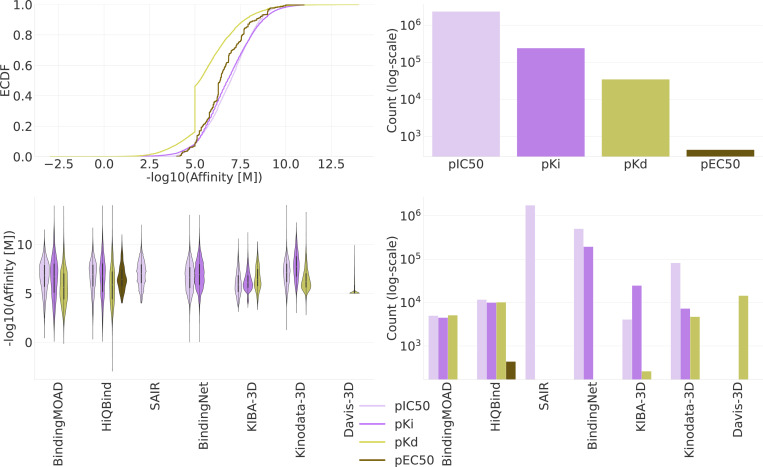
Overview of affinity data distributions and dataset coverage. **Top row:** (left) Empirical cumulative distribution functions (ECDFs) of affinity values for each affinity type across all datasets; (right) overall counts of each affinity type (log scale). **Bottom row:** (left) Distribution of affinity values per dataset and affinity type (horizontal violin plots); (right) affinity type counts per dataset (horizontal bar plots, log scale). Legend indicates affinity type color coding used throughout all plots.

**Figure 14:**
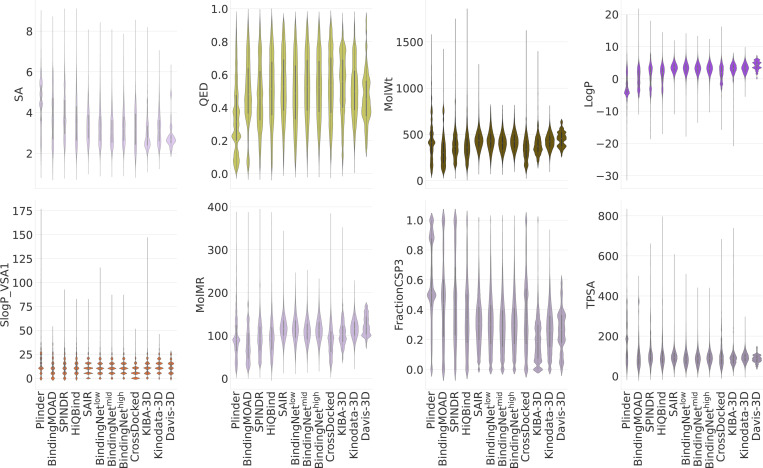
Comparison of key continuous molecular properties of ligands—including molecular weight, logP, molar refractivity, and topological polar surface area—across all datasets. The distributions reveal substantial variability in ligand size, polarity, and hydrophobicity, reflecting the chemical diversity and distinct selection criteria of each dataset.

**Figure 15:**
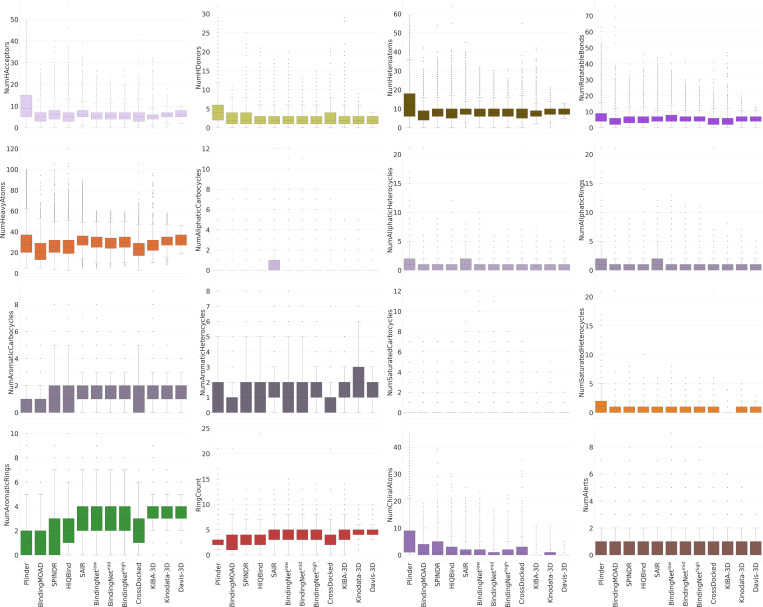
Comparison of the distributions of discrete ligand features such as hydrogen bond acceptors and donors, rotatable bonds, ring systems, and chiral centers for each dataset. The observed differences highlight variations in molecular complexity, flexibility, and functional group composition among the datasets, providing insight into their respective chemical spaces.

**Figure 16:**
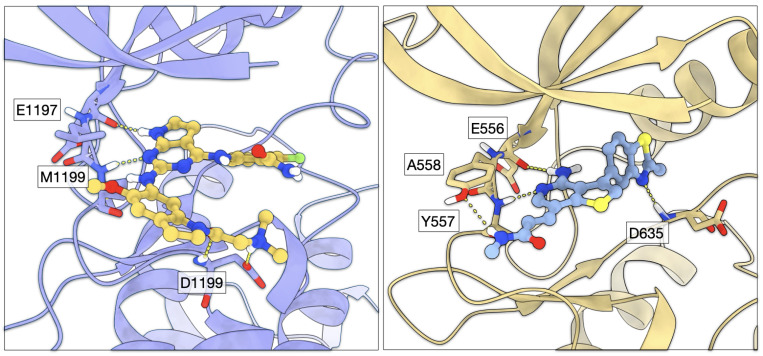
On the left, the structure of anaplastic lymphoma kinase (ALK, PDB ID: 2XB7, blue) with docked CHEMBL461876. The pyrrolopyrimidine moiety is positioned in the adenine region of the catalytic pocket, forming a bidentate hydrogen bond (yellow dashed lines) with the main chains of hinge residues Glu1197 and Met1199. On the right, the structure of the tyrosine kinase fibroblast growth factor receptor 3 (FGFR3, PDB ID: 6LVM, yellow) accommodates an aminopyridine derivative (CHEMBL1968590) in a similar manner, with a conserved bidentate H-bond pattern in the hinge region (Glu556 and Ala558), complemented by an additional H-bond to the Tyr557 side chain. Furthermore, the nitrogen of the thiazole ring forms a hydrogen bond with Asp635 from the functional DFG motif. Both poses were favourable scored, with Glide docking gscore below −11 (−11.4 for the 2XB7–CHEMBL461876 complex and −11.9 for the 6LVM–CHEMBL1968590 complex)

**Figure 17:**
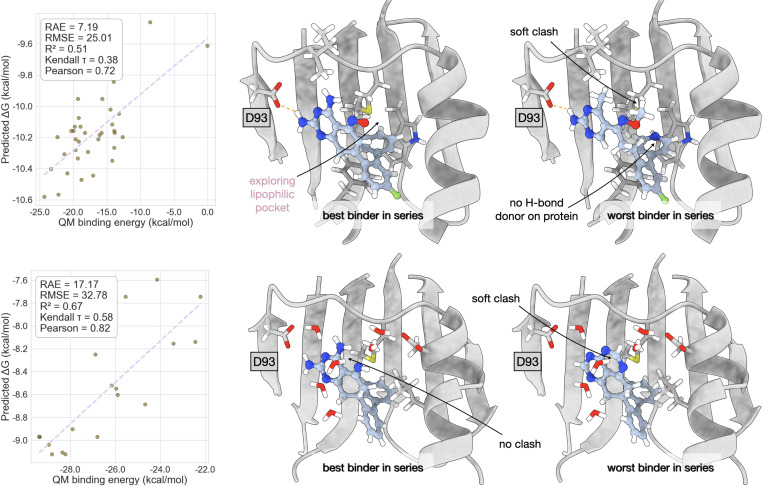
Flowr.root validation on Hsp90. **Top Block:** test1 validation, with more ligands but without explicit waters. **Left Block:** correlation between Flowr.root affinity metrics and QM binding energies. **Middle Block:** schematic representation of the best binder in the series. **Right Block:** schematic representation of the worst binder in the series. **Bottom Block:** test2 validation, with explicit waters considered in the QM calculations. **Left Block:** correlation between Flowr.root affinity metrics and QM binding energies. **Middle Block:** schematic representation of the best binder in the series. **Right Block:** schematic representation of the worst binder in the series.

## Data Availability

All datasets used in this study are publicly available and can be downloaded from Google Drive at https://drive.google.com/drive/folders/1NWpzTY-BG_9C4zXZndWlKwdu7UJNCYj8.

## Footnotes

1 Data from (08/2025) https://github.com/OpenFreeEnergy/IndustryBenchmarks2024

2 Results from (08/2025) https://blog.omsf.io/the-free-energy-of-everything-benchmarking-openfe

## Code availability

The source code for Flowr.root is publicly available on GitHub at github.com/jule-c/flowr_root. The pretrained model checkpoint can be downloaded from Google Drive at https://drive.google.com/drive/folders/1NWpzTY-BG_9C4zXZndWlKwdu7UJNCYj8.

## Appendix

### A Datasets

In Sec. 3 in Fig. 1 we visualize the data generation framework that we established for this work. As outlined in Sec. 3 we used as protein-ligand complex data Plinder, BindingMOAD, Spindr, HiQBind, SAIR, BindingNet, CrossDocked2020, KIBA-3D, Davis-3D, and Kinodata-3D. After downloading and aggregating the different data sources, we split proteins and ligands, if not already done, into separate files. We save ligands as SDF and proteins as PDB or CIF files. Afterwards, we filter the data and remove systems that classify the ligands either as ions, artifacts or not definable. Then, we use Schrodinger’s LigPrep to prepare the ligands and Schrodinger’s PrepWizard to prepare the protein-ligand complex (see Sec. 3 for more details). Successfully prepared systems are converted into our internal pocket-ligand representation encapsulated as PocketComplex instances for easy handling and data management. We retrieve pockets by cutting around the reference ligands with a cutoff of 7A and, if necessary, trim the pocket to the max size of 800 atoms. Every PocketComplex instance is annotated with comprehensive metadata spanning affinity values (if available), GNINA scores, interaction fingerprints, and selected RDKit features, like molecular weight, logP, TPSA, number of hydrogen acceptors and donors, number of rotatable bonds, number of aliphatic rings and many more. Finally, for all datasets we calculate distribution statistics comprising atom, charge, and bond type distributions as well as bond angles, bond lengths and dihedral angles distributions. Importantly, all datasets are splitted following the Plinder train, validation and test splits.

In Fig. 12, we compared the chemical composition of all protein–ligand datasets used in this work by analyzing the distributions of ligand atom types, protein atom types, and protein residue types. The aggregated distributions (top row) reveal the overall prevalence of each type, while the per-dataset heatmaps (bottom row) illustrate how these distributions vary between datasets. For atom types, a logarithmic scale was used to emphasize less frequent elements. Only the 20 standard amino acids were considered for residue type comparisons, and hydrogens were excluded from atom type analyses to improve interpretability.

To provide a comprehensive overview of the affinity data, Fig. 13 summarizes both the distribution and coverage of affinity types across all datasets. The ECDFs display the range and distribution of affinity values for each affinity type, highlighting differences in spread and modality. The overall affinity type counts, presented on a logarithmic scale, reveal a pronounced imbalance, with IC50 being the most abundant. Dataset-specific distributions are visualized using horizontal violin plots, while horizontal bar plots summarize the number of measurements for each affinity type within each dataset. The majority of affinity values originate from the SAIR and BindingNet datasets. Notably, BindingNet also provides Ki measurements, while HiQBind and BindingMOAD offer a balanced representation of IC50, Ki, and Kd values. HiQBind is the only dataset containing EC50 measurements. In contrast, Davis-3D is exclusively annotated with Kd values. This overview facilitates the identification of data biases and gaps that may influence downstream analyses.

To systematically assess the chemical diversity of ligands across datasets, we analyzed the distributions of several continuous molecular descriptors in Fig. 14, including molecular weight, logP, molar refractivity, and topological polar surface area (TPSA). These features capture key aspects of ligand size, hydrophobicity, polarizability, and polarity, which are relevant for molecular recognition and drug-likeness. The results reveal pronounced differences between datasets: for example, BindingNet and SAIR contain ligands with a broader range of molecular weights and higher TPSA values, while datasets such as HiQBind and BindingMOAD exhibit more constrained distributions. These trends reflect the varying selection criteria and source domains of the datasets, and highlight the importance of considering chemical diversity in benchmarking and model development.

We further characterized the datasets by comparing the distributions of discrete ligand features in Fig. 15, including the number of hydrogen bond acceptors and donors, rotatable bonds, ring systems, and chiral centers. These properties provide insight into molecular complexity, flexibility, and the presence of functional groups relevant for binding interactions. The analysis demonstrates that datasets such as BindingNet and SAIR encompass ligands with higher numbers of rotatable bonds and greater ring system diversity, whereas datasets like Davis-3D and KIBA-3D are more restricted in these aspects. Notably, the number of chiral centers and structural alerts also varies substantially, underscoring differences in stereochemical complexity and potential reactivity. Together, these comparisons elucidate the distinct chemical spaces sampled by each dataset and inform the interpretation of downstream modeling results.

#### A.1 KIBA-3D

The KIBA dataset was originally introduced by Tang *et al.* [Tang et al., 2014b] as a large-scale benchmark for kinase–ligand binding affinity prediction, integrating heterogeneous bioactivity readouts into a standardized KIBA score. The original dataset contained 52,498 chemical compounds, 467 kinase targets and over 240,000 interaction records. While comprehensive, the original KIBA dataset was highly scattered, due to an imbalance between the number of ligand-kinase pairs and associated measurements. To improve the density and ensure more reliable evaluation, subsequent studies discarded the ligands and targets with less than 10 interaction records. This procedure reduced the dataset to 2,094 ligands and 229 kinase targets. For this work, we prepared the 3D extension of KIBA (KIBA-3D) based on kinase targets with experimentally solved structures deposited in the PDB. Figure 15 shows examples of our docking results, illustrating that Glide could reproduce near-native binding poses for selected kinase inhibitors with conserved bidentate hydrogen bond pattern in the hinge region (e.g., PDBID:2XB7-CHEMBL461876, glide gscore −11,4, PDBID:6LVM-CHEMBL1968590, glide gscore −11,9; see Fig. 16. The KIBA-3D dataset serves as a large-scale, structure-consistent benchmark for evaluating generative and predictive models.

##### A.1.1 Target protein structure selection for KIBA-3D

Kinase targets from the KIBA dataset were mapped to their UniProt identifiers. For each UniProt accession, X-ray crystal structures were selected according to a set of heuristics to ensure biological relevance of the binding site. Only entries covering the kinase domain were considered. Among the available structures, the highest resolution one was chosen, with preference for holo over apo forms. Complexes with ATP, ADP, or staurosporine were deprioritized, as these ligands tend to stabilize non-representative conformations of the binding pocket; in particular, staurosporine, aside from being a highly promiscuous kinase inhibitor has a flat shape that inflate the catalytic site and biases the pocket toward accommodating larger ligands. Structures with fewer missing residues were favored, and cases where the bound small molecule occupied an allosteric or noncanonical pocket were excluded. The resulting curated set provides 174 kinase structures suitable for docking of the KIBA ligand set.

##### A.1.2 Docking

We performed docking with the Maestro suite (Schrödinger Release 2023–2: Maestro, Schrödinger, LLC, New York, NY, 2023) to generate consistent protein–ligand complexes across the 174 kinase targets. Small molecules were first optimized with LigPrep. All the 2094 ligands were processed. The dataset covers a broad chemical space, with molecular weights ranging from 180.15 to 1381.64 Da (median 376.49 Da). Protein structures were processed using the Protein Preparation Workflow, including protonation with Epik and propka [Johnston et al., 2023] and restrained minimization with the OPLS4 force field [Sastry et al., 2013]. Missing residues were built with Prime. Docking grids were centered on the kinase domain’s hinge region, covering the canonical ATP-binding pocket used by first-type kinase inhibitors. Docking was performed with the Glide SP protocol [Yang et al., 2021], with no constraints applied, generating one pose per ligand.

### B Additional results: Case studies

#### Hsp90: Heat shock protein 90

We also performed QM validation of Flowr.root’s affinity head prediction on the heat shock protein 90 (Hsp90), Figure 17. Unlike the cases in the main text, the dynamic range of predicted affinities is significantly smaller, a consequence of the narrower pocket filled with water molecules. We performed two sets of benchmarks, on different sets: test1 includes more ligands with higher variation in functional groups (Figure 17, top block): test2 includes explicit water in the QM calculations (Figure 17, bottom block). In all cases, critical interactions, like the hydrogen bond to Asp93, are retained over the ligand space. In test1, Flowr.root tried to explore interactions in a lipophilic subpocket of Hsp90 by replacing a terminal phenyl ring with a pyridyl, to realize the latter is less likely to be stabilized in the lipophilic environment. The model also replaced the pyrimidine ring with a quinazoline, in order to reduce the ligand’s degrees of freedom, leading to less penalizing changes in entropy upon binding. This is seen when comparing the worst and best binders of the series. Finally, Flowr.root also tried to explore substitutions on the pyrimidine/quinazoline rings to further grow the ligand. As this is a solvent-exposed domain, *NH*_2_ groups are favored and lead to better interaction patterns. In the case of test2, we explicitly included water molecules in the QM calculations, on top of implicit aqueous environment. This resulted in significantly better correlation between Flowr.root and the QM binding energies, although some of the poorer ligands had poorer QM scores due to soft clashes with the water molecules. This indicates that, although Flowr.root implicitly learns the composition and orientations of solvation layers, future work might involve giving the model the ability to capture these dynamically.
